# Tailed Lytic Bacteriophages of Soft Rot Pectobacteriaceae

**DOI:** 10.3390/microorganisms9091819

**Published:** 2021-08-26

**Authors:** Konstantin A. Miroshnikov, Peter V. Evseev, Anna A. Lukianova, Alexander N. Ignatov

**Affiliations:** 1 Shemyakin-Ovchinnikov Institute of Bioorganic Chemistry, Russian Academy of Sciences, Miklukho-Maklaya Str., 16/10, 117997 Moscow, Russia; petevseev@gmail.com (P.V.E.); a.al.lukianova@gmail.com (A.A.L.); 2 Timiryazev Agricultural Academy, Russian State Agrarian University, Timiryazevskaya Str., 49, 127434 Moscow, Russia; an.ignatov@gmail.com; 3 Department of Biology, Lomonosov Moscow State University, Leninskie Gory, 1, bldg. 12, 119234 Moscow, Russia; 4 Agrobiotechnology Department, Agrarian and Technological Institute, RUDN University, Miklukho-Maklaya Str., 6, 117198 Moscow, Russia

**Keywords:** bacteriophage, *Pectobacterium*, *Dickeya*, taxonomy, morphology, genomics, phage control, adsorption, tail spike protein

## Abstract

The study of the ecological and evolutionary traits of Soft Rot Pectobacteriaceae (SRP) comprising genera *Pectobacterium* and *Dickeya* often involves bacterial viruses (bacteriophages). Bacteriophages are considered to be a prospective tool for the ecologically safe and highly specific protection of plants and harvests from bacterial diseases. Information concerning bacteriophages has been growing rapidly in recent years, and this has included new genomics-based principles of taxonomic distribution. In this review, we summarise the data on phages infecting *Pectobacterium* and *Dickeya* that are available in publications and genomic databases. The analysis highlights not only major genomic properties that assign phages to taxonomic families and genera, but also the features that make them potentially suitable for phage control applications. Specifically, there is a discussion of the molecular mechanisms of receptor recognition by the phages and problems concerning the evolution of phage-resistant mutants.

## 1. Introduction

Modern agriculture experiences substantial difficulties with the treatment and prevention of diseases of staple plants caused by phytopathogenic bacteria [1]. Particularly, bacteria comprising the genera *Pectobacterium* and *Dickeya*, regarded as Soft Rot Pectobacteriaceae (SRP), are known to be causative agents of aerial rot, soft rot and blackleg in potato, cabbage, corn and other crops and in ornamental plants [2]. The use of protective chemicals is strictly limited and often ineffective. Therefore, protective measures are limited to quarantine and the control of seed material health [1]. The biological control of bacterial diseases has been an important topic in recent decades. An advanced approach in biocontrol is the use of specific bacteriophages, bacterial viruses which regulate bacterial populations in the environment. The history of phage control in plant science is long. The first applications against *Xanthomonas* and *Pectobacterium* (then *Erwinia*) spp. were developed in the 1920s, soon after the discovery of bacteriophages. Modern applications of phage control of phytopathogenic bacteria are reviewed in [3,4].

The taxonomic diversity of SRP is considerable. Previously uniformly regarded as “pectolytic *Erwinia*”, the genera *Pectobacterium* and *Dickeya* currently comprise about 30 separate species [5]. Such a multiplication of the taxons is mainly based on genomic features, thanks to the constant growth of available data of whole-genome sequencing and advanced pipelines for phylogenomic analysis [6].

Even more dramatic changes in taxonomy currently occur for bacteriophages and viruses in general. Viral taxonomy has acquired a classic Linnean 15-rank pyramidal structure [7,8], raising philosophical questions about the place of viruses in live nature [9]. Taxonomic attribution of bacteriophages based on genomic features has been attempted previously [10] and is currently used as a major rule [11], disregarding classic definitions of phages that are based on morphological properties.

Taking this into consideration, it is cumbersome to define a newly characterised bacteriophage, as well as to attribute previously described phages in accordance with updated taxonomy. The available information on complete genomes of bacteriophages is growing very rapidly [12]. For example, the first review of phages of *Pectobacterium* and *Dickeya* spp. was published in 2015 and described 20 phages [13]. Currently, the NCBI GenBank database contains 108 genomic sequences of phages infecting *Pectobacterium* and *Dickeya* spp. (as accessed on 15 June 2021). However, a recent review devoted to SRP very briefly described corresponding bacteriophages [5]. Thus, one of the purposes of the current review is to fill the informational gap in this important area.

One of the major disadvantages of phage therapy in medicine and phage control in agriculture is the excessively high specificity of phages. The terms “broad” and “narrow” when applied to a host range of a particular phage are somewhat speculative (discussed in [14]). Most known phages infect only a few strains within a bacterial species and phages with a host range covering several related species are rare. Thus, the creation of ample phage panels containing comprehensively characterised phages infective to all abundant strains of the target pathogen is a necessary step in the preparation of phage cocktails.

According to the general rules for the composition of such cocktails [15,16], it is preferable to use distinct phages with different molecular mechanisms of infection. This reasonable requirement, preventing or reducing the formation of phage-resistant mutants of target bacteria, is not easy to fulfil. It is generally considered that phage-resistant mutants of bacteria are less virulent than the wild type [17,18]. However, the pathways used by bacteria to protect the population from phage attack are numerous and diverse, from the spatial escape of motile bacteria to molecular mechanisms of inactivation of the infecting phage and programmed bacterial death before phage multiplies [14,19,20,21,22]. Some aspects of the functioning of toxin–antitoxin [23,24] and CRISPR-Cas [25,26,27,28] systems of bacterial anti-phage protection have been studied in detail with respect to *Pectobacterium* sp. An important means of acquiring phage resistance is the modification or loss of the surface receptor that the bacteriophage uses for infection. However, this action reduces the fitness and/or virulence of the resulting phage-resistant bacteria. Therefore, the composition of phages using different receptors of the same target bacteria increases the effect of the phage cocktail, simultaneously reducing the evolution of resistant mutants. The knowledge of what molecule on the bacterial surface serves as a phage receptor helps to rationalise the combining of phages. Where available, the authors will provide information concerning phage receptors.

An important area of investigation is the presence of integrated phage sequences in bacterial genomes. Almost all sequenced SRP genomes contain mobile elements where genes originating from bacteriophages can be recognised [29,30,31]. Genomic information enables the prediction that integrated phages (prophages) can represent all three morphologies of tailed phages [29]. Prophages may be inducible, and phage transfer from the lysogenic infectious cycle to a lytic one plays an important role in the regulation of the bacterial population. It is worth noting that the first wave of interest in temperate phages of SRP evolved in the 1970s. Then, the *Erwinia carotovora* strain 268 was used as a biotechnological producer of asparaginase in the USSR, and production was often hindered by the spontaneous activation of prophages with the subsequent lysis of bacterial cells. Early studies on the repertoire of temperate phages contained within this strain, conditions of prophage induction and the primary characterisation of phage particles and DNA were performed at the Zabolotny Institute of Microbiology and Virology in Kiev [32,33,34]. Another notable phage studied in the same Institute, in post-Soviet Ukraine, is ZF40, a temperate *Pectobacterium carotovorum* phage, which was extensively used to study generalised transduction and lysogeny. ZF40 is one of the most comprehensively studied representatives of the new “dwarf” Myovirus group [35,36]. A further interesting example is the *Dickeya dadantii* (then, *E. chrysanthemi*) phage ϕEC2, which was isolated in the early 1980s [29]. This phage was widely used in studies of generalised transduction and genome mapping [37,38,39], but the sequence of this phage is unavailable. Generally, information about temperate phages of SRP is fragmentary and undetailed. In the context of phage therapy/control applications, temperate phages are considered to be undesirable due to the potential transfer of genomic fragments and alteration of the properties of lysogenised strains [40]. Therefore, the discussion of temperate phages is beyond the scope of the present review.

## 2. Bacteriophages Infecting *Dickeya* and *Pectobacterium* spp.: Overview and Classification

### Principles of Genomic Comparison

**Figure 1:**
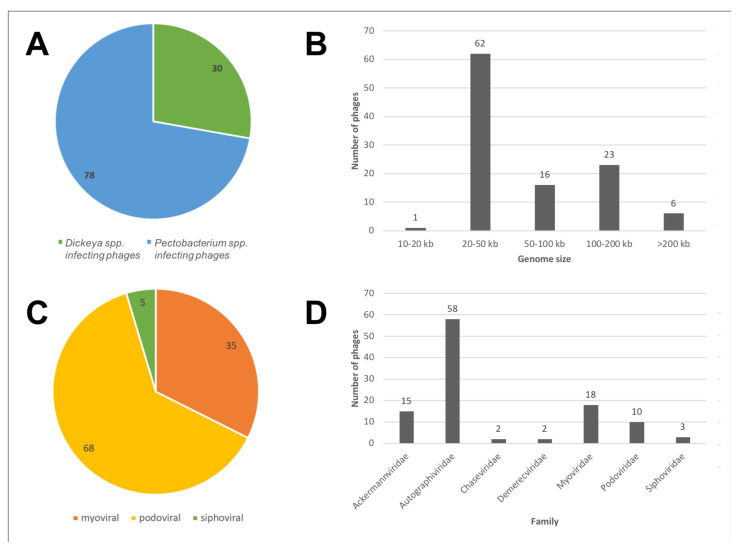
Statistics on 108 SRP bacteriophages’ genomes deposited in the GenBank genome database, as of June 2021. (**A**) SRP phages’ host affiliation; (**B**) Genome size distribution; (**C**) Morphology of phages; (**D**) Taxonomic affiliation at the level of family.

The history of genomic investigations of SRP bacteriophages is relatively short. The first complete genomes of phages infecting *D. solani* [41] and *P. carotovorum* [42] were published in 2012. Since SRP belong to the order *Enterobacterales*, all identified phages of *Pectobacterium* and *Dickeya* spp. have analogs among phages infecting more commonly characterised enterobacteria (*Escherichia*, *Salmonella*, *Klebsiella,* etc.). However, the metabolic peculiarities of SRP adapted to different environmental conditions and diverse natural factors affect the diversity of corresponding phages and their genomic properties. As of 15 June 2021, 108 complete and partial genomes of SRP bacteriophages had been published in the GenBank genome database (Supplementary Table S1). They belong to seven families of the order *Caudovirales* and comprise a wide variety of phages, including the tailed bacteriophage DU_PP_III, with the smallest known genome of 11.5 kb and a Jumbo phage vB_PcaM_CBB with a genome of 378 kb [43]. Most of them (68 phages) represent phages of Podoviral morphology; 30 phages infect *Dickeya* and 78 phages infect *Pectobacterium* hosts (Figure 1).

**Figure 2:**
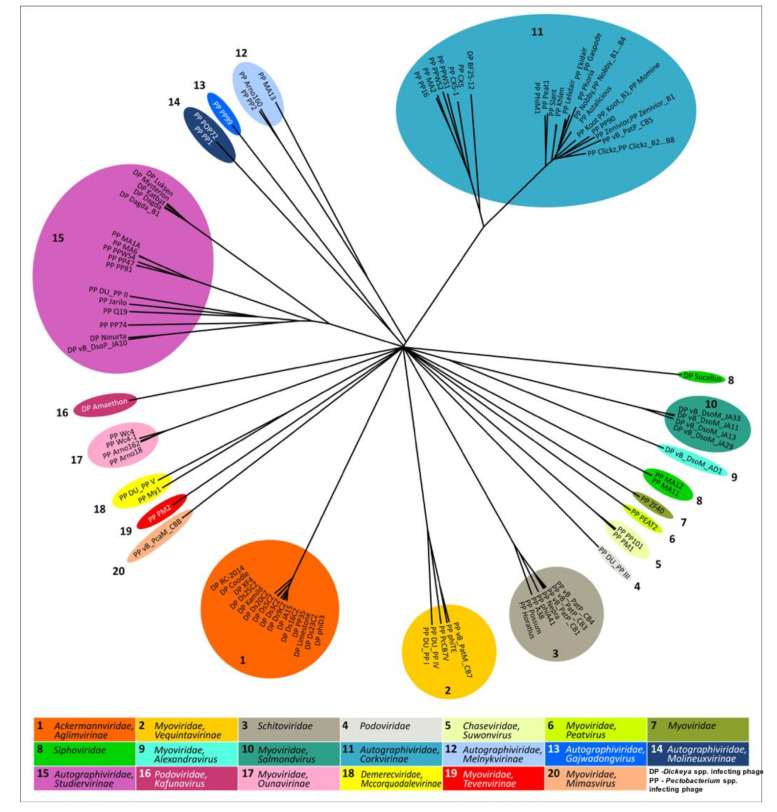
The tree representing the BIONJ clustering based on the VIRIDIC intergenomic similarity values of 108 GenBank SRP bacteriophage genomes. The phage taxonomy is indicated in the captions.

To assess the taxonomy and evolutionary relations between bacteriophages, the most conventional way is the alignment of complete genomic sequences. Intergenomic similarity can be evaluated with various online tools and stand-alone software packages, which perform the calculations of ANI (Average Nucleotide Identity) or other whole-genome comparisons (ANI calculator Kostas lab [44], OrthoANI [45], Gegenees [46], JSpecies [47], Mauve [48], VICTOR [49], VIRIDIC [50], etc.). The latter, the VIRIDIC (Virus Intergenomic Distance Calculator) tool, is often preferable, since it is built on the traditional BLASTN method used by the Bacterial and Archaeal Viruses Subcommittee (BAVS) from the International Committee on Taxonomy of Viruses (ICTV), to evaluate viruses’ intergenomic relatedness. This pipeline was used in the present work with “-word_size 7 -reward 2 -penalty -3 -gapopen 5 -gapextend 2” settings. The dendrogram representing the results of the VIRIDIC clustering was constructed with Phylogeny.fr [51] using the BIONJ algorithm [52]. The dendrogram, based on the VIRIDIC intergenomic similarity values of all GenBank SRP phage genomes, and visualised using Geneious Prime 2021.2.2 [53], is shown in Figure 2. The tree clusters all the phage taxa in distinct clades but does not clearly show the relations at the level of subfamily and higher.

The applicability of the analysis based on the whole genomes can be limited because of extensive horizontal exchanges and the dissimilar evolutionary history of proteins belonging to the same phage [54,55,56]. Another problem is fast sequence drift [57] hampering the consistency of alignments. Even close phage species can possess unique ORFs. To clarify the evolutionary relations between phages, whole-genome comparisons should be supplemented by other methods.

Each species of bacterial host results in the evolutionary adaptation of GC composition and an assortment of used codons in phage genomes. Thus, the alignment and comparison of marker genes unique to bacteriophages are desirable for robust phylogenetic estimation. Viruses (including bacteriophages) contain no ribosomes. Therefore, the 16S rRNA gene, universal for all living organisms, is not applicable for this purpose. The search for marker genes that are universal for all bacteriophages of a particular high-ranking group (order, phylum) but have enough difference in sequence to be characteristic for lower-ranking taxonomic types (genus, subfamily) is challenging.

Alignments using the sequences of the marker genes discussed below were made with MAFFT 7.48 [58,59] using the L-INS-I algorithm and default settings. The alignments were trimmed with trimAL [60], with -gappyout settings. The best protein model was estimated with ModelTest-NG [61]. The phylogenetic analysis was conducted with the Bayesian inference of phylogeny by MrBayes [62,63] and with RAxML 8.2.10 [64,65], using the GAMMA LG protein model. The robustness of the MrBayes trees was assessed by estimating the average standard deviation of split frequencies and posterior probability. The robustness of the RAxML trees was assessed by the bootstrap analysis.

The gene encoding major capsid protein (MCP) is the conventional object for comparing viruses with an icosahedral proteinaceous capsid. These proteins have a common spatial architecture for all icosahedral viruses of bacteria, archaea, plants and animals [66]. Therefore, differences in the size and sequence of MCP genes reflect the evolution of these genes and may be suitable for drawing phylogenetic conclusions. The phylogenetic tree based on the MCP amino acid sequences of SRP bacteriophages is shown in Supplementary Figure S1.

RNA polymerase (RNAP) complex is an essential component of the genome of many bacteriophages. To optimise the transcription of the viral genome in the course of infection, phages encode one, or several, of their own RNAPs. These polymerases may be significantly different from similar enzymes of the host bacterium. Comparison of the sequences of phage RNAPs will, thus, reflect the evolutionary relations of these phages. Sequences and genomic locations of promoter sites enabling the functioning of phage RNAP are also important parameters describing the phylogenetic positioning of the studied phage.

The packaging of the genomic DNA into the capsid is an important stage of morphogenesis for all tailed bacteriophages (phylum *Uroviricota*, order *Caudovirales*). It provides the ability for infection of the newly formed phage particle. The key role in this process is played by terminase, one of the most conservative phage proteins [67]. This complex consists of 1–3 subunits encoded in the phage genome. The difference in composition and sequence of terminase proteins may serve as a characteristic of the evolutionary position of the phage. At the level of genera and sometimes higher, the topology of the phylogenetic tree based on the amino acid sequences of the large (ATPase) subunit of terminase of SRP bacteriophages (Supplementary Figure S2) resembles the topology of the MCP tree (Supplementary Figure S1). It might be suggested that the MCP and terminase trees show the relations between phage taxa better than the BIONJ VIRIDIC tree (Figure 2).

**Figure 3:**
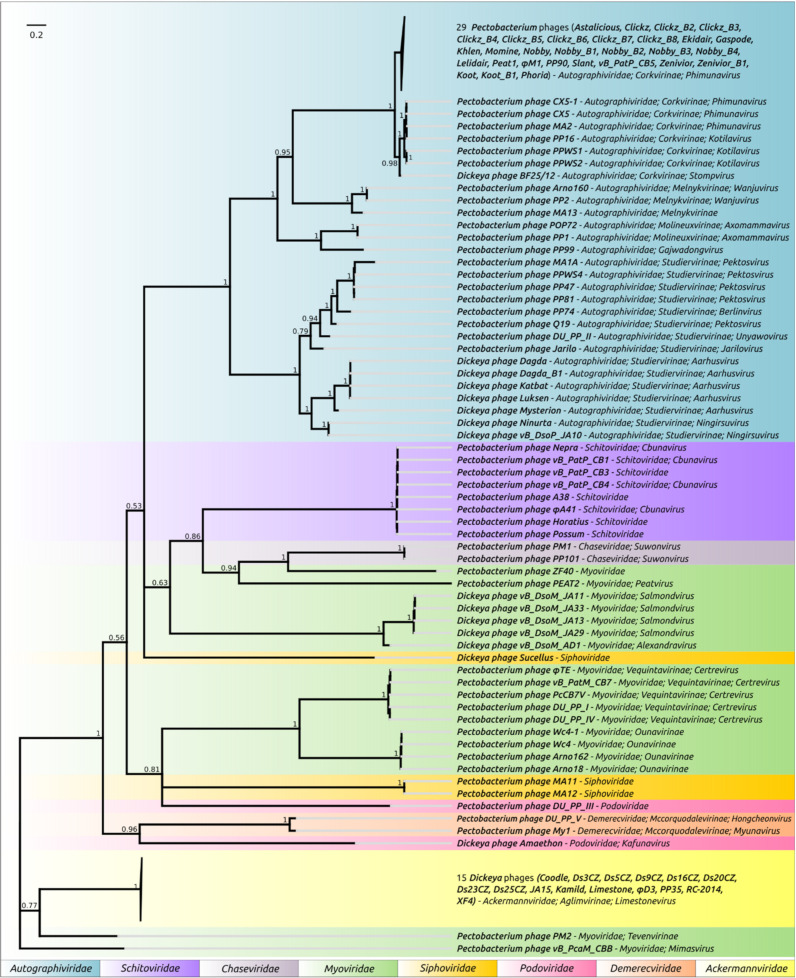
Phylogenetic tree obtained with MrBayes based on concatenated amino acid sequences of major capsid protein and terminase large subunit. Bayesian posterior probabilities are indicated near their branches. Taxonomic classification is shown to the right of the phage name. The scale bar shows 0.2 estimated substitutions per site and the tree was rooted to *Pectobacterium* phage vB_PcaM_CBB. The chain length was 3,300,000, the burn-in length was 300,000, the subsampling frequency was 200 and the average standard deviation of split frequencies was 0.0106.

The concatenation of sequences of marker genes can result in more reliable conclusions [68]. However, the reliability of concatenated sequences’ phylogeny can be compromised when the genes have contradictory evolutionary histories [69]. The tree constructed using concatenated amino acid sequences of MCP and terminase belonging to 107 SRP bacteriophages, where those sequences were presented, is shown in Figure 3. The concatenated sequences tree demonstrates the phylogenetic relations of proteins at the level of phage subfamilies and, in some cases, families.

Fine phylogenetic positioning of the phage also often considers the sequences of the genes encoding the adsorption apparatus of the phage and the lysis of the host bacteria at the end of the infection cycle. Further description of taxonomic groups (families, subfamilies or genera) of bacteriophages infecting *Pectobacterium* and *Dickeya* spp. will shed light on the patterns and details related to the characteristic genes listed above.

The division of bacteriophages into groups according to the morphology of virus particles determined by transmission electron microscopy, being transformed to taxonomic names, was conventionally used for the description of phages [35,70,71]. Tailed phages belonging to each characteristic morphogroup—Myoviruses with a long contractile tail, Podoviruses with a short expandable tail and Siphoviruses with a long flexible non-contractile tail—share a critical feature in terms of how the phage delivers genomic DNA into the host cell and the conformational changes in tail proteins that accompany this process. Therefore, on the subject of the structural organisation of tailed phages, even the most recent textbooks use the morphological criterion [72]. The unified naming of viruses of microorganisms provided in 2008, (e.g., T4 = vB_EcoM_T4, where vB—virus of bacteria, Eco—abbreviation of the bacterial host *E. coli*, M, P or S denotes morphology and the last letters name the virus), is still in use [73]. On the other hand, it was shown that genomic differences were more important for the taxonomic definition of phages. However, currently, only some groups of phages have been found to be worthy of attribution as a separate family. Most others are still listed as subfamilies and separate genera of the families *Myoviridae*, *Podoviridae* and *Siphoviridae* based on morphology. Thus, given that modern taxonomic requirements are based on genomics, it is useful to consider phage morphology as one of the most important characteristics, at least until a non-systematic definition is adopted.

## 3. Morphotype Myoviridae

### 3.1. The Ackermannviridae Family

The discovery of numerous and closely related phages infecting *Pectobacterium* and *Dickeya*, now belonging to the family *Ackermannviridae*, was closely linked to the outbreak of a newly emerged phytopathogen, *Dickeya solani*, in the mid-2000s. This SRP species was possibly formed from a strain group of *D. dianthicola* [74] which demonstrates pronounced virulence to potatoes. The focused isolation of phages infective to *D. solani* became a tendency in applied phage biology during the 2010s. It is curious that the first isolated *D. solani* phage*,* Limestone [41], and many other phages isolated in Europe, were closely related and belonged to a type not previously associated with *Pectobacterium* and *Dickeya* phages. A systematic search of *D. solani* phages has revealed the prevalence of phages of this type in the environment [75,76].

**Figure 4:**
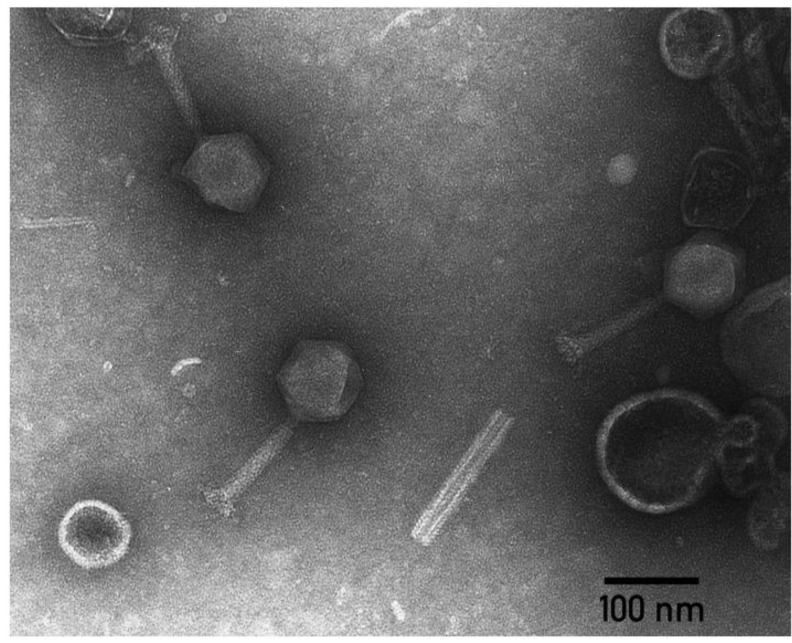
Transmission electron microscopy of *Ackermannviridae Dickeya* phage PP35 [80]. The scalebar is 100 nm. The image was kindly provided by Dr. Ekarterina Obraztsova.

The morphology of the described *D. solani* phages is typical of A1 Myoviruses, with an icosahedral capsid 90–100 nm in diameter, a 120–140 nm-long contractile tail and an extended base plate complex decorated with ~10 nm-long tail spikes (Figure 4). The most closely related phage at the time was *Salmonella* phage Vi1 [77], so Limestone-like phages were attributed to genus *Vi1virus * [78]*. *According to the current taxonomic distribution, this group of *Dickeya* phages belongs to the genus *Limestonevirus*  of the subfamily *Aglimvirinae*, family *Ackermannviridae* [79].

Currently, the NCBI GenBank contains 15 genomes of *Limestonevirus* bacteriophages, the primary isolation host for all of them being *D. solani* (Table 1). The type phage of the genus Limestone [41] has been thoroughly studied genomically and proteomically. Therefore, it is possible to identify the functions of a substantial part (up to 30%) of genes. Generally, the genomes of Limestone-like phages have a large size (150–156 kbp) and possess a clustered bidirectional organisation known for T-even bacteriophages. Genomes of all *Limestonevirus* phages encode a single tRNA.

Many elements of gene regulation, such as the sequences and locations of promotors governing the transcription of gene cascades, are conservative in all *Aglimvirinae* phage genomes. Another typical genomic feature is hypermodified pyrimidine residues derived from 5-hydroxymethyl-2′-deoxyuridine (5-hmdU). These modified residues have been experimentally identified for phage Vi1 [84]. Genes responsible for 5-hmdU transformation, dUMP hydroxymethyl transferase, a-glutamyl/putrescinyl-thymidin phosphotylase and kinases are strictly conservative among all *Ackermannviridae* phages.

A noticeable genomic feature of *Aglimvirinae* phage genomes (including *Limestonevirus*) is the presence of multiple genes of homing endonucleases in genomes. The number of these genes varies from 14 to 25 and their location in the genome may be one of identifying features of a particular Limestone-like phage [83]. The exact contribution of homing endonucleases in the phage lifecycle is not clear. However, there have been some indications that enzymes of this type can promote horizontal gene transfer of surrounding genes when two related phages co-infect the same host [85]. Analysis of *Limestonevirus* phage genomes infecting SRP shows the presence of numerous random sequences differing in G+C composition from the rest of the genome [86,87].

Non-specific transduction in Limestone-like phages has been shown experimentally [76]. Thus, despite the probability of these events being considered to be low, it is advisable to be cautious when using phages of this type therapeutically, particularly in field trials.

All *Ackermannviridae* phages have a highly conservative gene cluster encoding a complex of the baseplate and adsorption apparatus. This operon involves about 10 genes, where a noticeable difference is observed in the sequences of tail spikes only. N-terminal domains of tailspike proteins (TSP) responsible for the attachment of the tail spike to phage particles are almost identical in all *Ackermannviridae* phages, independent of the infection range of the phage. Tail spikes participate in the primary adsorption of the phage particle to the bacterial cell interacting with the molecules on the cell surface [88].

Structural investigations of recombinant TSP of phages infecting *Enterobacteria* [89,90], *Pseudomonas* [91] and *Acinetobacter* [92] have revealed the fairly uniform composition of these complexes. TSP backbone is formed by a trimeric β-helix. Domains interacting with cell receptors may be located on the surface of the trimeric prism or within the loops protruding from the prism [93]. If bacterial surface polysaccharide (O-polysaccharide, OPS or capsular polysaccharide, CPS) serves as a primary receptor for the phage, TSP may contain enzymatic domains degrading or modifying it [94]. The sequences of C-terminal TSP domains are almost identical in *Ackermannviridae* bacteriophages infecting *D. solani* only. These parts of polypeptide chains are responsible for binding OPS. Since *D. solani* is a recently evolved pathogen, the diversity of OPS is low. It is possible that all *D. solani* strains have the same OPS structure inherited from the “parent” strain of *D. dianthicola* [95]. It has been demonstrated experimentally that recombinant TSP of phage PP35 splits OPS into 8–10 carbohydrate unit fragments [80]. Two *Limestonevirus* phages, ϕPD10.3 and ϕPD 23.1,  have been reported to infect *P. parmentieri* (*wasabiae*) and *P. carotovorum*, as well as *D. solani* [87]

In comparison to essential medical pathogens like *E. coli* [96], information concerning surface polysaccharides of SRP is fragmentary. Structures of OPS of some strains of *Pectobacterium* sp. [97,98,99,100,101,102] and *Dickeya* sp. [80,95,103,104] have been revealed by NMR, and they were found to be very diverse. It is reasonable to propose that additional tailspikes should provide recognition of OPS with different structures. Genomic analysis indicates the presence of an additional TSP gene in the genomes of ϕPD10.3 and ϕ PD 23.1. The C-terminal sequence of the second TSP is shorter and differs from one known for degrading *D. solani* OPS, but also contains a predicted enzymatic domain (GenBank accession numbers KM209229—KM209273 for the draft genome of phage ϕ PD10.3   and KM209274—KM209320 for ϕ PD23.1). Bacteriophages are known for the presence of several different tail spikes/fibres, with different specificity, expanding the host range among related bacteria [105,106]. In the case of SRP, the metabolic difference between the species, and even genera, may be small enough to provide efficient infection of various strains by a phage recognising several different surface receptors. This feature can give such phages an environmental and evolutionary benefit. If one ignores the problem of generalised transduction typical of *Ackermannviridae* phages (see above), the search for, and selection of, such multihost phages could be a reasonable solution for the purposes of SRP phage control.

### 3.2. The Chaseviridae Family

**Figure 5:**
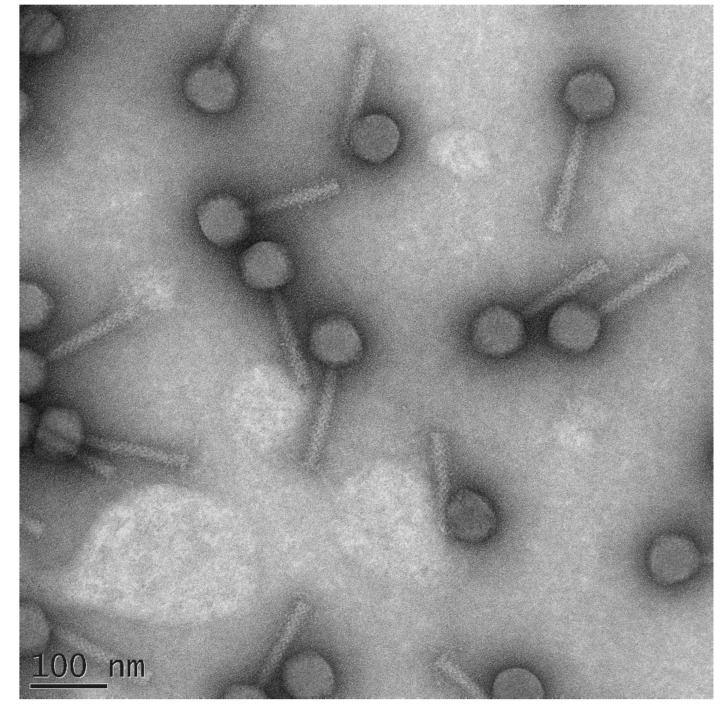
Transmission electron microscopy of *Pectobacterium* phage PP101 [102]. The scalebar is 100 nm. The image was kindly provided by Dr. Ekarterina Obraztsova.

The establishment of this family was proposed by ICTV in 2019 and ratified in 2021. *Chaseviridae* have united a large group of lytic Myoviruses with isometric capsids 55–65 nm in diameter, a thin neck and 110–130 nm-long tails (Figure 5). The first representative of this family, *Escherichia* phage GJ1, was isolated and sequenced in the mid-2000s [107]. Broad host range of GJ1 lysing many enterotoxigenic *E. coli* strains has attracted special attention [108]. More recently, similar phages have been identified for other Gammaproteobacteria. Genomes of these phages are 50–55 kbp in size and are circularly permuted, with long direct repeats. Encoded ORFs show similarity with both Myoviral and Podoviral proteins, and the distinctive feature is RNA polymerase very similar to T7-like phages (*Autographiviridae*).

*Chaseviridae* RNAP is located early in the genome, provides unidirectional transcription and uses promotors with a similar sequence and arrangement through the phage genome. The family is divided into two subfamilies: *Cleopatravirinae*, comprising *Escherichia, Erwinia, Pectobacterium, Pantoea* and *Proteus* phages, and *Nefertitivirinae*, which includes *Shewanella* and *Aeromonas* phages. The first phage infecting *P. carotovorum*, PM1 [109], was isolated in Korea and became a type phage for the genus *Suwonvirus*. Later this genus was accompanied by another species, PP101 [102]. The infection range of phage PP101 has been shown to be relatively broad, covering the strains of *P. versatile* and *P. brasiliense* [102], raising questions about the principle of how *Chaseviridae* phages recognise their hosts. All sequenced phages of the family have orthologs of three proteins annotated as tail fibre proteins in PP101. It has been proposed that *Chaseviridae* phages have multicomponent tail fibres resembling those of long-tail fibres of phage T4 [110]. This hypothesis has no experimental proof, except for the visible complex tail fibres in a high-resolution EM image of the phage Y2 infecting *Erwinia* [111], currently attributed as a member of subfamily *Cleopatravirinae*, family *Chaseviridae*, and the presence of a putative distal component of the tail fibre, Y2 gp86, in the structural proteome of the phage [111]. The adsorption mechanism and bacterial receptors of phages comprising this new family need further investigation, but, if confirmed, this tripartite composition of tail fibres may be one of the hallmark features of *Chaseviridae* phages.

### 3.3. The Vequintavirinae Subfamily

The establishment of subfamily *Vequintavirinae* was proposed by ICTV in 2015 and ratified in 2016. According to the latest ICTV taxonomic updates, the subfamily comprises five genera, of which the *Certrevirus* genus contains the SRP bacteriophage *Pectobacterium* phage ϕTE. There are also four phages in the GenBank genome databases attributed as belonging to the *Certrevirus* genus (Table 2), but the taxonomic classification of these phages appears to need clarification. The VIRIDIC matrix points to the intergenomic similarity between some phages in Table 2 being slightly lower than the regular genus threshold of 70% (Supplementary Figure S3). However, these phages are similar in genome layout and biological features, and the terminase phylogeny places the phages in a distinct clade (Supplementary Figure S4). Phages DU_PP_I and DU_PP_IV can be considered as a clonal group.

Transmission electron microscopy has shown that phages ϕTE and CB7 possess an A1 myoviral morphology with a capsid of approximately 94 nm diameter (ϕTE) and 84 nm (CB7) and a tail of about 120 nm in length [112,113].

The *Vequintavirinae* SRP phages have a relatively large genome of more than 140 kbp, encoding up to 260 proteins. Four out of five genomes, except for ϕTE, encode the tRNA genes. The genomes are characterised by a significant number of HNH endonuclease genes, which might be related to the splitting of several genes, including DNA polymerase, terminase and others. A comparative genomic analysis has demonstrated that the phages conserved strategies of DNA replication, DNA metabolism, host lysis and virion structure [113].

The phage CB7 has a limited host range, infecting only the isolation host strain *P. atrosepticum* DSM 30,186 and four other *P. atrosepticum* strains, and the phage is ϕTE capable of causing generalised transduction [113], which should be taken into account in relation to its use in phage therapy.

### 3.4. The Ounavirinae Subfamily

This subfamily, established in 2016, is represented by four phages of an unclassified genus (Table 3). The phage Wc4-1 was obtained through adaptive evolution to elevated temperature, from the ancestral Wc4. Their genomes are nearly identical, except for a single nucleotide substitution. Phage Wc4 demonstrates a Myoviral morphology, with an icosahedral capsid ~58 nm in diameter and a contractile tail of 97 nm in length [114].

The genomic comparisons (the VIRIDIC matrix is shown in Supplementary Figure S5) and the terminase large subunit phylogeny (Supplementary Figure S6) indicate that all four bacteriophages are closely related, enabling them to be assigned to a distinct single genus of the *Ounavirinae* subfamily.

The phage genomes have a similar size of around 92 kbp and a similar GC content of 44.5–44.7%, which is somewhat lower than for typical *Pectobacterium* genomes. The genomes contain 24 tRNA genes for 19 amino acids and are characterised by a large number of orphane genes, which account for about 90% of all genes. However, the sequence search and HMM-HMM comparison demonstrate the presence of typical phage DNA-polymerase I and several genes encoding the nucleic acids’ metabolism proteins.

Phage Wc4 has been efficiently tested both alone and as part of a phage cocktail against 20 *P. carotovorum* strains [115]. The adapted phage Wc4-1 showed better stability when subjected to heat treatment at 60 °C for 1 h and after 60 days of storage at 37 °C while being identical to the wild-type ancestral phage in infectivity and lytic properties [114].

### 3.5. The Tevenvirinae Subfamily

Bacteriophage T4 infecting *E. coli* is a traditional model object of molecular virology. The history of T4-related research exceeds 70 years, and much is known about all aspects of T4’s structure and functioning. T4-like phages recently attributed as members of subfamily *Tevenvirinae* are widespread in the biosphere. In the early 2000s, it was proposed that the sequence diversity of the marker gene *g23* encoding MCP of T4-like phages could be used to assess the ecology of viroplankton [116]. This proposition is debatable, since no T4-like phages are known for many bacterial families, but it reflects an attitude to the phage most studied for that moment. *Tevenvirinae* phages are considered suitable for phage therapy, and several reports of therapeutic cocktails comprising T4-like phages only are available [117,118,119].

The history of the structural study of phages infecting *Pectobacterium* and *Dickeya* spp. (*Erwinia*, at the time) started with an image of a viral particle with a morphology characteristic of T4, which was presented in the very first paper in which electron microscopy was used to characterise these objects [120]. An electron microscopy image of Myophage with an elongated, T4-like capsid was shown for another unsequenced phage infecting *P. carotovorum* [121]. Some mutants of the classic phage T4 were shown to infect Enterobacteria that were different from *E. coli*, including *Erwinia*/*Pectobacterium* spp. This observation enabled researchers to study the role of lipopolysaccharides (LPS, the main receptor of phage T4) in pectobacterial pathogenicity [122]. However, the search for T4-like phages specifically infecting *Pectobacterium* and *Dickeya* spp. yielded very few results. The number of sequenced *Tevenvirinae * phages infecting SRP has been limited to a single phage, PM2, isolated by a Korean group of scientists [123]. This lytic phage is infectious to field isolates attributed as *P. carotovorum* and *P. brasiliense.* Genomic studies have indicated that PM2 is a T4-like phage possessing a genome of 178 kbp with typical architecture and a high level of homology of most essential ORFs with other *Tevenvirinae* phages, RB69 and JS98.

An adsorption apparatus is similar in architecture to phage T4. It contains both a complex of long-tail fibres (PM2_gp 270–273), where the distal part is much shorter than that of phage T4, and short-tail fibres (PM2_gp 181). Therefore, it is reasonable to hypothesise that the adhesion apparatus of PM2 uses a similar set of LPS and porins, like LamB and OmpC in *E. coli* and other enterobacteria [124,125], to infect a bacterial host. These molecules have a limited diversity, which explains the relatively broad host range of PM2 covering the strains of several species of *Pectobacterium*.

### 3.6. The Mimasvirus Genus

**Figure 6:**
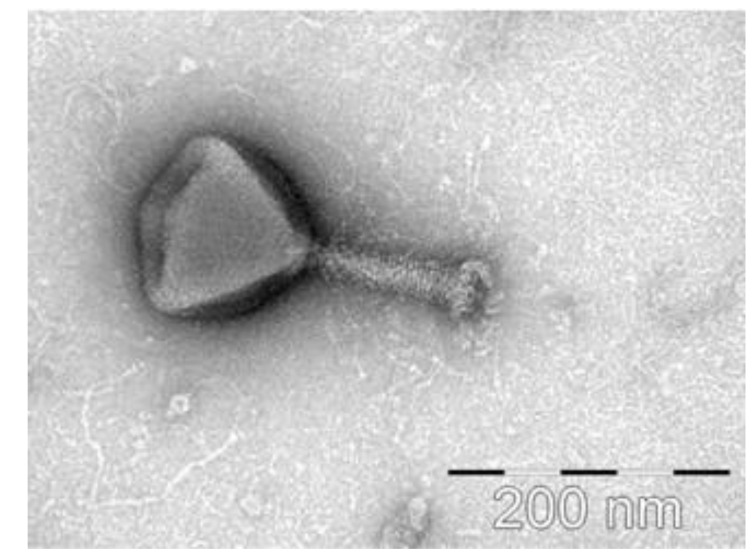
Transmission electron microscopy of *Pectobacterium* phage vB_PcaM_CBB. The scalebar is 200 nm. The image was kindly provided by Dr. Colin Buttimer and Dr. Aidan Coffey.

This genus is represented by *Pectobacterium* (*Enterobacteria)* phage vB_PcaM_CBB (Figure 6), which exhibits activity against a broad range of hosts, including species belonging to the genera *Erwinia*, *Pectobacterium* and *Cronobacter* [43].

CBB has one of the biggest phage genomes, of 355,922 bp, containing 554 ORFs, and features long, direct terminal repeats of 22,456 bp. A comprehensive bioinformatic analysis [43] including genomic comparison and phylogenetic studies found similarities with so-called “RAK2-like phages”, a group of phages which includes *Klebsiella* (*Enterobacteria)* phage vB_KleM-RaK2 and other Jumbo phages belonging to the *Alcyoneusvirus*, *Asteriusvirus*, *Eneladusvirus* and *Mimasvirus* genera.

CBB phage possesses a typical A1 myoviral morphology, with an isometric head 123 nm in diameter and a contractile tail 128 nm in length. The phage seems to be characterised by a complicated adsorption apparatus comprising long-tail fibres and numerous spike-like structures [43]. The complexity of the CBB virion is reflected in a proteome comprising more than 80 structural proteins. Another interesting feature of the CBB genome is the presence of genes presumably involved in translation. The list of these genes comprises 33 tRNA genes for 21 amino acids, including pyrrolysine-tRNA, tRNA processing genes and translation initiation factor IF-3.

The broad host range of *Pectobacterium* phage vB_PcaM_CBB presents an interesting perspective for phage therapy/control.

### 3.7. The Alexandravirus and Salmondvirus Genera

The genus *Alexandravirus* is represented by the *Dickeya* phage vB_DsoM_AD1. This jumbo phage has a genome of 262 kbp, containing 330 ORFs. The phage infects only *D. solani*, on which it was isolated [126]. The phage virion has a capsid with a diameter of 120 nm, a tail 150 nm in length and structures at the base of the tail not clarified by electron microscopy [126]. Phylogenetic studies and genomic analyses have indicated the relatedness of vB_DsoM_AD1 and phages JA11, JA13, JA29 and JA33 discussed further, to a voluminous group of large phages also containing the genera *Baikalvirus*, *Mieseafarmvirus*, *Salmondvirus* and *Yoloswagvirus* [126,127].

The genus *Salmondvirus* is represented by four similar phages infecting *Dickeya* species [126,128]. A summary of these phages is shown in Table 4. These phages are characterised by a genome about 250 kbp in size, encoding around 320 proteins. The micrograph of phage JA29 [126] shows it to be similar to phage CBB, the “hairy Myoviridae” morphology [43,126], with an isometric head approximately 130 nm in diameter and a tail approximately 170 nm in length. All four phages were found to lyse *D. solani*, *D. dadantii subsp. dieffenbachiae* and *D. paradisiaca*. In addition, phages JA11, JA13 and JA33 were found to be capable of infecting *D. dianthicola* and *D. zeae*. Phage JA29 adsorbs to species other than *D. solani*, but with 10^−4^ less efficiency, while phages JA11, JA13 and JA33 can adsorb to all species of *Dickeya* with similar efficiency [126].

*Alexandravirus* and *Salmondvirus* phages have similar genome structures. An interesting feature of these phages (as well as *Baikalvirus* and *Yoloswagvirus*) is their possession of two distinct tail sheath proteins. Phylogenetic analysis has demonstrated that these proteins seem to have arisen from a common predecessor by gene duplication after the divergence of *Mieseafarmvirus* [127,128].

### 3.8. The Peatvirus Genus

*Pectobacterium* phage PEAT2 (GenBank accession no. MG432137) was isolated using *P. atrosepticum* as a host, in 2019 [129]. The phage was assigned to the *Peatvirus* genus comprising this phage only (ICTV ratification in March 2020). According to electronic microscopy images [129], PEAT2 possesses an isometric-headed Myoviral A1 morphotype.

The phage has a genome of 48,659 bp and a GC-content of 49.1%. The genome contains 55 ORFs and does not contain tRNA genes. Genomic analysis suggests that this Myovirus has a lytic infectional cycle. A VIRIDIC intergenomic comparison (Supplementary Figure S7) suggests a close relatedness to *Jedunavirus* phages (intergenomic similarity values are 56–60% compared to *Klebsiella* phages belonging to this genus). More distant relatives are *Pantoea* phage AAM22 (MK 798142) rated as unclassified *Myoviridae,* and *Edwardsiella* phages belonging to the genus *Yokohamavirus* of the *Myoviridae* family. The terminase phylogeny confirmed these relationships, placing PEAT2, *Jedunavirus* phages, *Pantoea* phage AAM22 and *Yokohamavirus* phages in a distinct clade (Supplementary Figure S8).

## 4. Morphotype Podoviridae 

**Figure 7:**
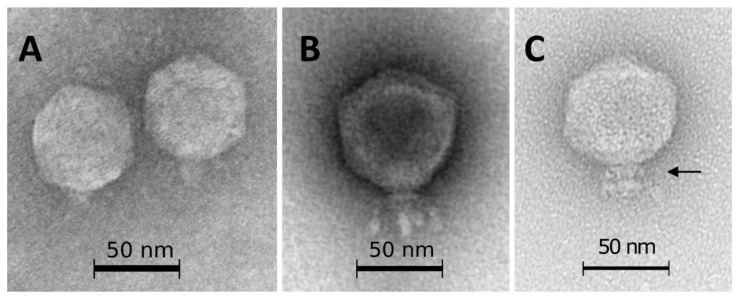
(**A**) Transmission electron microscopy of *Pectobacterium* phage Arno160 (*Autographiviridae* family) [101], (**B**) *Pectobacterium* phage vB_PatP_CB4 (*Schitoviridae* family) [130], (**C**) *Dickeya* phage Amaethon (*Kafunavirus* genus); (**C**) [101]. The scale bar is 50 nm. The arrow points to the collar structure beneath the capsid. The images were obtained with the kind permission of the authors and publishers of the cited papers.

The list of SRP bacteriophages contains 68 phages with Podoviral morphology belonging to two families (*Autographiviridae* and *Schitoviridae*) and one genus (*Kafunavirus*). The micrographs of the *Pectobacterium* phage Arno160 (*Autographiviridae* family) [101], *Pectobacterium* phage vB_PatP_CB4 (*Schitoviridae* family) [130] and *Dickeya* phage Amaethon (*Kafunavirus* genus) [131], representing these taxa, are shown in Figure 7. All three phages possess the C1 morphotype with an icosahedral capsid of about 60 nm (Arno160), 70 nm (vB_PatP_CB4) and 67 nm (Amaethon) in size, and distinct collar structures beneath the capsid. The phage Arno160 has a short tail about 10 nm in length, with tail spikes attached [101]. The phage vB_PatP_CB4 (and close phages CB1 and CB3) has a 25 nm tail and a set of short (25 nm) whiskers attached to a collar structure, with whiskers ending with elongated globular appendices at their distal end [130]. The phage Amaethon also has short-tail fibre appendages beneath the collar structure [131].

### 4.1. The Autographiviridae Family

*Autographiviridae* bacteriophages comprise the majority of SRP phages with published genomes. They include 58 phages belonging to *Corkvirinae*, *Melnykvirinae*, *Molineuxvirinae* and *Studiervirinae* subfamilies and to the *Gajwadongvirus* genus. Most SRP *Autographiviridae* phages belong to the *Studiervirinae* subfamily, consisting of *sensu lato* T7-like phages. Because of their virulent lifestyle, comparatively short life cycle and a big burst size, *Autographiviridae* bacteriophages are promising as components of phage cocktails.

**Figure 8:**
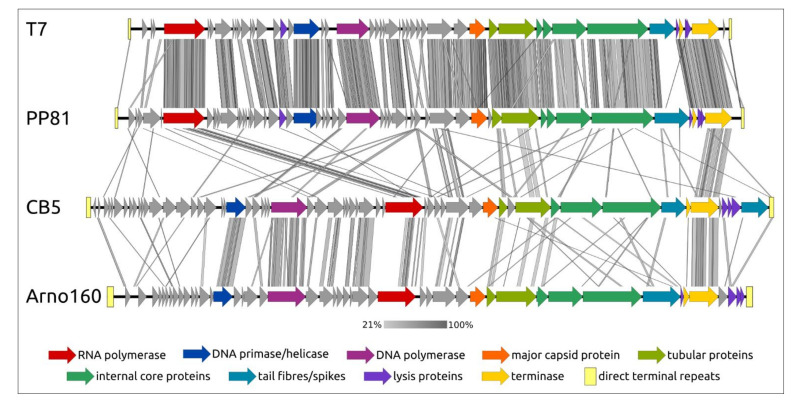
Genome comparison between *Autographiviridae* phages made with EASYFIG and TBLASTX. T7—*Escherichia* phage T7 (*Studiervirinae*, *Teseptimavirus*), PP81—*Pectobacterium* phage PP81 (*Studiervirinae*, *Pektosvirus*), CB5—*Pectobacterium* phage vB_PatP_CB5 (*Corkvirinae*, *Phimunavirus*), Arno160—*Pectobacterium* phage Arno160 (*Melnykvirinae*, *Wanjuvirus*). The percentage of sequence similarity is indicated by the intensity of the grey colour. Vertical blocks between analysed sequences indicate regions with at least 21% similarity.

The structure of genomes of SRP *Autographiviridae* phages belonging to different taxa has much in common. Meanwhile, the sequences of the majority of proteins except for the large subunit of terminase, major capsid protein and several transcriptional and replication genes can differ substantially (Figure 8).

The genomes of all *Autographiviridae* phages comprise unidirectional genes and can be divided into three major functional regions: the early region, associated with host conversion, DNA metabolism region and morphogenesis regions. The early genome regions of *Pectobacterium* phages belonging to the *Studiervirinae* and *Molineuxvirinae* subfamilies and the *Gajwadongvirus* genus contain T7-like, DNA-dependent RNAP genes. In phage T7, RNA polymerase plays an exceptionally important role in participating in the transcription of phage genes, in phage genome replication making primers for use by DNA polymerase and in genome packaging [132,133,134]. Interestingly, in the genomes of phages belonging to the *Melnykvirinae* and *Corkvirinae* subfamilies, the RNAP gene is located closer to the middle of the genomes (Figure 8). It is also noteworthy that genomes of SRP *Autographiviridae* phages seem to contain the gene of transfer RNA nucleotidyltransferase (TRNT), the CCA-adding enzyme, significantly more often than other *Autographiviridae* phages [135]. The BLAST search revealed no homologs of *Autographiviridae* phage TRNP among the proteobacterial sequences but indicated the presence of distantly related proteins in *Aquificae*, *Bacteroidetes*, *Spirochaetia*, *Actinobacteria* and *Terrabacteria* bacteria, including thermophilic organisms. The HHpred analysis (toolkit.tuebingen.mpg.de) indicated the high level of structural similarity of the *Autographiviridae* TRNP with eukaryotic mitochondrial CCA-adding enzymes. It seems that the *Autographiviridae* phages acquired this protein at the beginning of their evolutionary history, and, for some reason, the genomes of SRP bacteriophages are prone to keep TRNP genes.

The adsorption apparatus of *Autographiviridae* SRP bacteriophages can include T7-like tail fibres or tail spikes, which possess an enzymatic activity, degrading the host OPS or assisting in adsorption, or having both of these impacts [101,102,135,136,137,138]. The phages can exchange the modules of adsorption proteins with the participation of bacterial hosts [139], and this process has been shown to happen for *Autographiviridae* phages infecting *Pectobacterium* hosts [136].

**Figure 9:**
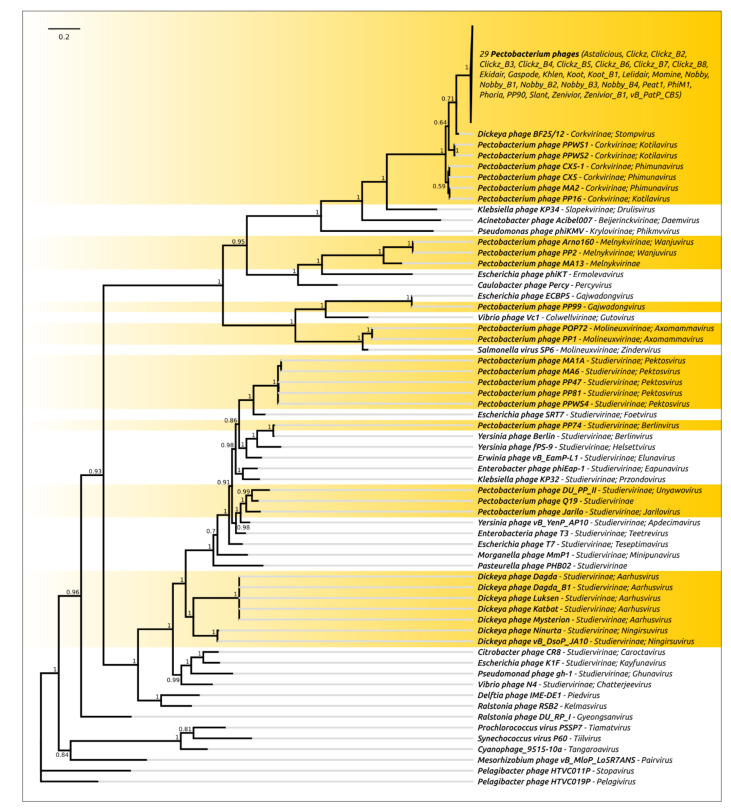
Phylogenetic tree obtained with MrBayes, based on the terminase large subunit amino acid sequences of *Autographiviridae* phages. Bayesian posterior probabilities are indicated near their branches. Taxonomic classification is shown to the right of the phage name. The scale bar shows 0.2 estimated substitutions per site and the tree was rooted to *Pelagiabacter* phage HTVC011P. The chain length was 3,300,000, the burn-in length was 300,000, the subsampling frequency was 200 and the average standard deviation of split frequencies was 0.0077. Eight monophyletic SRP branches are highlighted.

The VIRIDIC intergenomic comparison (Supplementary Figure S9) and terminase phylogeny (Figure 9) indicate that even closely related *Autographiviridae* SRP bacteriophages may have a distinct origin. At the moment, they can be divided into eight monophyletic lines. Line 1 comprises the majority of the SRP *Autographiviridae* phages, the phages belonging to the *Corkvirinae* subfamily. This group includes 32 *Pectobacterium* phages of the *Phimunavirus* genus, three *Pectobacterium* phages of *Kotilavirus* genus and *Dickeya* phage BF25/12 representing the *Stompvirus* genus. Line 2 comprises three *Pectobacterium* phages belonging to the *Melnykvirinae* subfamily, including two phages of the *Wanjuvirus* genus and one unclassified phage. Line 3 comprises only one phage, *Pectobacterium* phage PP99, which is assigned to the *Gajwadongvirus* genus. Line 4 includes two *Pectobacterium* phages belonging to the *Axomammavirus* of the *Molineuxvirinae* subfamily. The situation in the *Studiervirinae* subfamily is more complicated. The terminase phylogeny points to the occurrence of several monophyletic lines of the *Studiervirinae* SRP phages. Line 5 includes nine *Dickeya* phages of the *Aarhusvirus* and *Ningirsuvirus* genera, Line 6 contains *Pectobacterium* phage PP74 of the *Berlinvirus* genus, Line 7 comprises five *Pectobacterium* phages of the *Pektosvirus* genus and Line 8 includes two *Pectobacterium* phages belonging to the *Jarilovirus* and *Unyawovirus* genera and unclassified *Pectobacterium* phage Q19.

SRP *Autographiviridae* phages are promising as biocontrol agents. There were indications that treatment with both one phage and phage cocktails decreased tissue maceration and lesions because of soft rot disease, with an efficacy up to 98% [140,141,142,143,144,145]. Some phages (Jarilo, PP47, Q19, etc.) have a relatively broad host range [135,138], while others demonstrate a narrower host range (Arno160, CB5) [101,136]. It is probable that the use of multiple host strains for phage isolation can result in an increase in the frequency of phages with a broad host range [146]. However, there can be a trade-off between the host range and the efficiency of phage infection. The use of cocktails composed of a mixture of different phages promotes diminishing negative consequences of this trade-off.

### 4.2. The Schitoviridae Family

The *Schitoviridae* family encompasses some 120 N4-like bacteriophages with a genome organisation similar to the *Escherichia* phage N4. The family was recently proposed by Wittmann et al. [147] and ratified by the ICTV in March 2021 [148]. Four bacteriophages infecting the *Pectobacterium* species are assigned to the genus of *Cbunavirus* of the *Schitoviridae* family in the ICTV Master Species List [149], but an analysis of the GenBank sequences indicates that more SRP phages can be assigned to the *Schitoviridae* family (Table 5).

**Figure 10:**
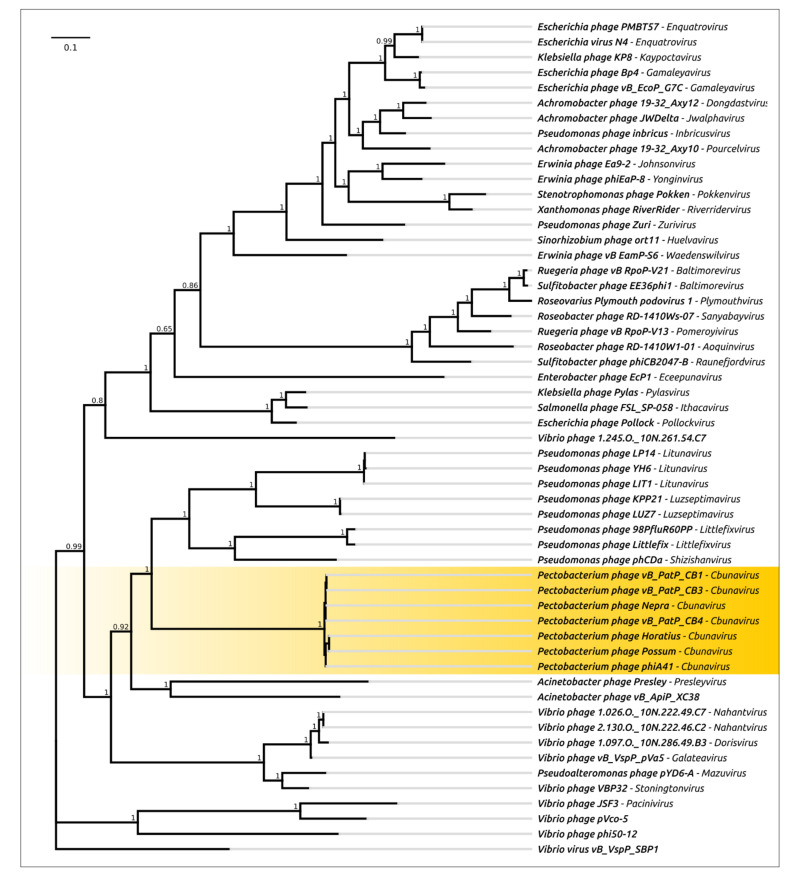
Phylogenetic tree obtained with MrBayes, based on the terminase large subunit amino acid sequences of *Schitohiviridae* phages. Bayesian posterior probabilities are indicated near their branches. Taxonomic classification is shown to the right of the phage name. The scale bar shows 0.1 estimated substitutions per site and the tree was rooted to *Vibrio* phage vB_VspP SBP1. The chain length was 2,200,000, the burn-in length was 200,000, the subsampling frequency was 200 and the average standard deviation of split frequencies was 0.0017. The SRP bacteriophage clade is highlighted.

The results of an intergenomic comparison indicated that all these phages can be assigned to the *Cbunavirus* genus, demonstrating a VIRIDIC intergenomic similarity of 79.4% and higher (Supplementary Figure S10). A phylogenetic analysis using the concatenated sequences of a major capsid protein and a large subunit of terminase indicated the closeness of the phages and the monophylicity of the branch comprising all *Pectobacterium* N4-like phages (Figure 10). This analysis, as well as the tree based on the DNA-polymerase and virion RNA polymerase, published earlier [130], points to *Acinetobacter* phage Presley, *Litunavirus* and *Luzseptimavirus* phages as being close groups in terms of their evolutionary history. The CG content of all the phages is very similar (48.5–48.7%) and is typical for the majority of SRP phages. The frequency of G and C nucleotides is lower for the early region of the genome and higher for the structural genes. Six of the eight *Pectobacterium* N4-like phages contain one or two complete tRNA genes (tRNA-Asn and/or tRNA-Gln), but the sequence search demonstrated the presence of complete and/or partial sequences of both tRNA genes in all eight *Cbunavirus* genomes.

**Figure 11:**
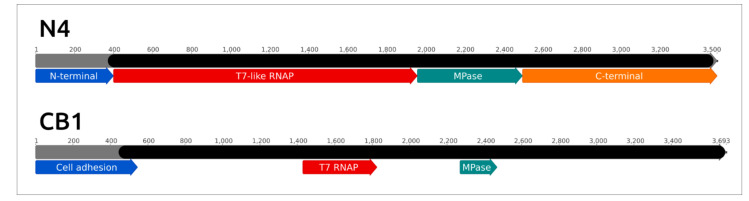
The scheme illustrating the positions of protein domains in the primary sequences of vRNAP of *Escherichia* phage N4 (upper scheme) and *Pectobacterium* phage vB_PatP_CB1 (lower scheme). The scale shows the position of amino acid residues starting from the N-end of the protein. The parts of N4 and CB1 vRNAPs coloured black are similar, according to the HHpred analysis. The parts of N4 vRNAP coloured blue, red, green and orange indicate the positions of N-terminal, T7-like RNAP, zincin-like metalloprotease and C-terminal domains, respectively, according to [154]. The parts of vB_PatP_CB1 vRNAP coloured blue, red and green indicate the positions of structurally similar parts of proteins, including the cell adhesion catenin alpha-1 (PDB structure 4K1N, Phyre2 confidence 99.5%), T7 RNAP (PDB structure 1MSW, Phyre2 confidence 93.5%) and metalloprotease PPEP-2 (PDB structure 6FPC, Phyre2 confidence 92.5%).

A notable feature of the *Schitoviridae* phages is the presence of three genes encoding RNAP [151,152]. Two genes encode two subunits of RNAP II, responsible for the transcription of middle and late genes [153,154]. The transcription of early genes is processed by encapsulated virion RNA-polymerase (vRNAP), which is injected into the host cell from the beginning of infection [151,155]. vRNAP is encoded by the largest gene of the phage genome. vRNAP appears to be a polyvalent multifunctional protein [156]. It comprises an N-part, which is important for injection into the host, a central part that hosts RNA-polymerase activity and is structurally similar to T7 RNAP and a C-part that is essential for the encapsidation of protein [157,158,159]. A thorough bioinformatic analysis indicated the more complex structure of vRNAP, which can vary between different *Schitoviridae* phages [156]. In particular, the N4 vRNAP includes a zincin-like metallopeptidase domain located between the T7-like RNAP domain and the C-terminal domain (Figure 11). The sequence comparison of *Cbunavirus* virion RNA-polymerases indicated a high level of similarity between the proteins belonging to different *Pectobacterium* phages (with an amino acid pairwise identity of 97.5% and higher). The *Cbunavirus* vRNAP amino acid sequence does not have a high pairwise identity with the N4 RNAP, but the HMM analysis demonstrated the overall similarity between the CB1 and N4 vRNAPs, except for the N-terminal part. It seems that the *Cbunavirus* vRNAP has the same domain structure as the N4 vRNAP, including the presence of the zincin-like metallopeptidase domain, which was confirmed by the HMM search (Figure 11).

The adsorption proteins of all *Cbunavirus* phages comprise at least one long (about 900 aa) tail fibre protein that is similar for all eight phages (pairwise identity of about 80% and higher). Primary sequence and HMM-HMM comparisons have indicated that these tail fibres possess a short N-terminal conservative T7-like domain, which can bind to the phage particle, and a central SGNH hydrolase-type domain. In phage CB4, the tail fibre is possibly encoded by two adjacent genes. The genomes of *Cbunavirus* phages contain a gene encoding a short 240 aa length conservative protein. According to HHpred analysis results, the N-part of this protein is structurally similar to the N-part of phage λ’s tail fibre and the C-part slightly similar to the T7 tail fibre part, including residues 118–209, (the length of the T7 fibre protein is about 570 aa). The high conservatism of this protein compared to the long fibre protein, and the absence of structural similarity with the receptor-binding domains of proteins of other phages, might indicate that this protein could perform structural, rather than receptor-binding, functions.

The host range of many N4-like phages infecting *Escherichia*, *Pseudomonas*, *Vibrio* and *Roseovarius* spp. is limited to the strain used in the original isolation [130,160,161,162], probably because of the fairly complicated mechanism for the establishment of phage infection, which requires the efficient injection of vRNAP. However, the host range of phages CB1, CB2 and CB4 was relatively broad within their *P. atrosepticum* host species, collectively infecting 15 of the 19 *P. atrosepticum* strains tested [130]. The host range of phage Nepra is restricted by *P. atrosepticum* strains and partially overlaps with CB1, CB3 and CB4 host strains [145]. The phages ϕA38 and ϕA41 were able to infect 6 of the 21 *P. parmentieri* isolates tested [150]. It seems that the potency of *Schitoviridae* phage therapy can be weakened by the limited host range, but these phages can be used in biocontrol applications as a part of phage cocktails.

### 4.3. The Kafunavirus Genus

The NCBI database contains six genomes of phages attributed as members of the *Kafunavirus* genus, including phage Amaethon, which has been isolated using *D. dadantii* strain *NCPPB 4097* (Figure 7C) [131]. The Amaethon genome shows GC-content of 39.8%, which is significantly lower than the genome GC-content of sequenced *D. dadantii* strains (about 56%). This difference enables the suggestion that either *D. dadantii* is not a natural host of the phage, or the history of their host–parasite relationship is relatively short. The Amaethon genome contains 49 ORFs and only 25 genes were found to have homologs in genomes of other *Kafunavirus* phages [131].

The VIRIDIC intergenomic comparison has also shown the limited similarity between Amaethon and *Kafunavirus* phages (Supplementary Figure S11). Applying the 70% genome similarity genus threshold, it is possible to assign the phages currently attributed as *Kafunavirus* to four distinct genera. Actually, according to the ICTV master species list, only one phage, the *Edwardsiella* virus KF1, is officially classified as a representative of the *Kafunavirus* genus (ratification in February 2019). The taxonomy of the phages related to KF1 possibly needs further revisions.

The large subunit terminase and major capsid protein phylogeny (Supplementary Figures S12 and S13) indicate the relatedness of these proteins with homologous proteins encoded in the genomes of temperate phages and bacterial prophage regions. It is probable that the ancestry of the *Kafunavirus* genome formation includes the participation of temperate phages in the relatively recent past.

The sequence search indicated the presence, in the *Dickeya* phage Amaethon genomes, of a gene encoding a tail spike protein that seems to comprise the phage adsorption apparatus. The Amaethon tail spike is very similar to the tail spike of *Dickeya Limestonevirus* bacteriophages but is less similar to proteins of other phages and is dissimilar to *Kafunavirus* phage proteins. It is possible that it was obtained with the participation of horizontal transfer.

### 4.4. Pectobacterium Phage DU_PP_III

**Figure 12:**
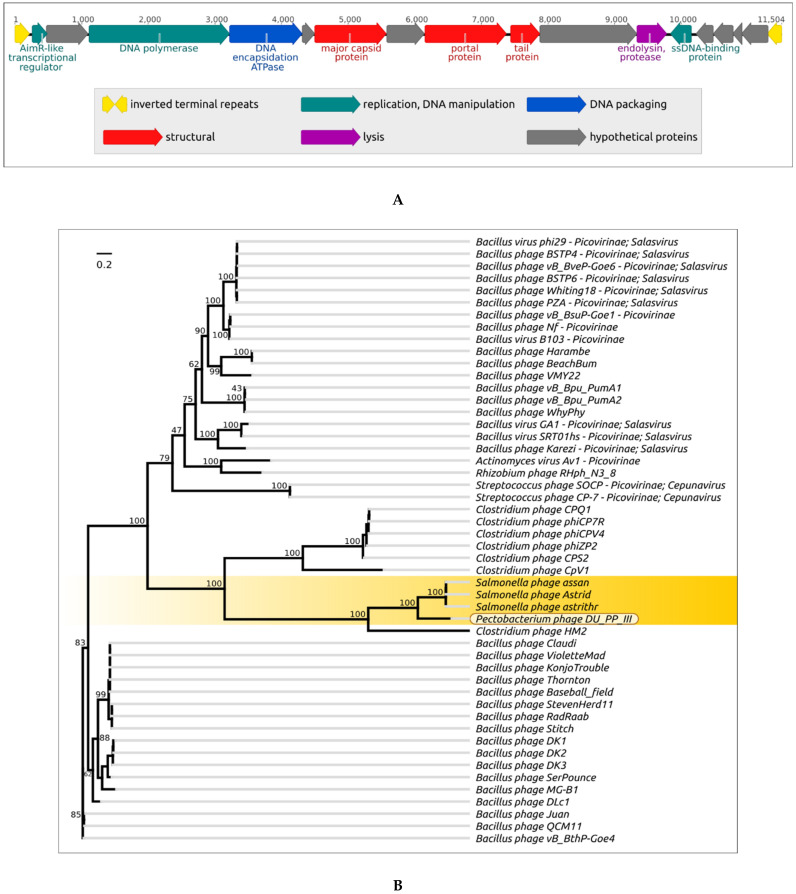

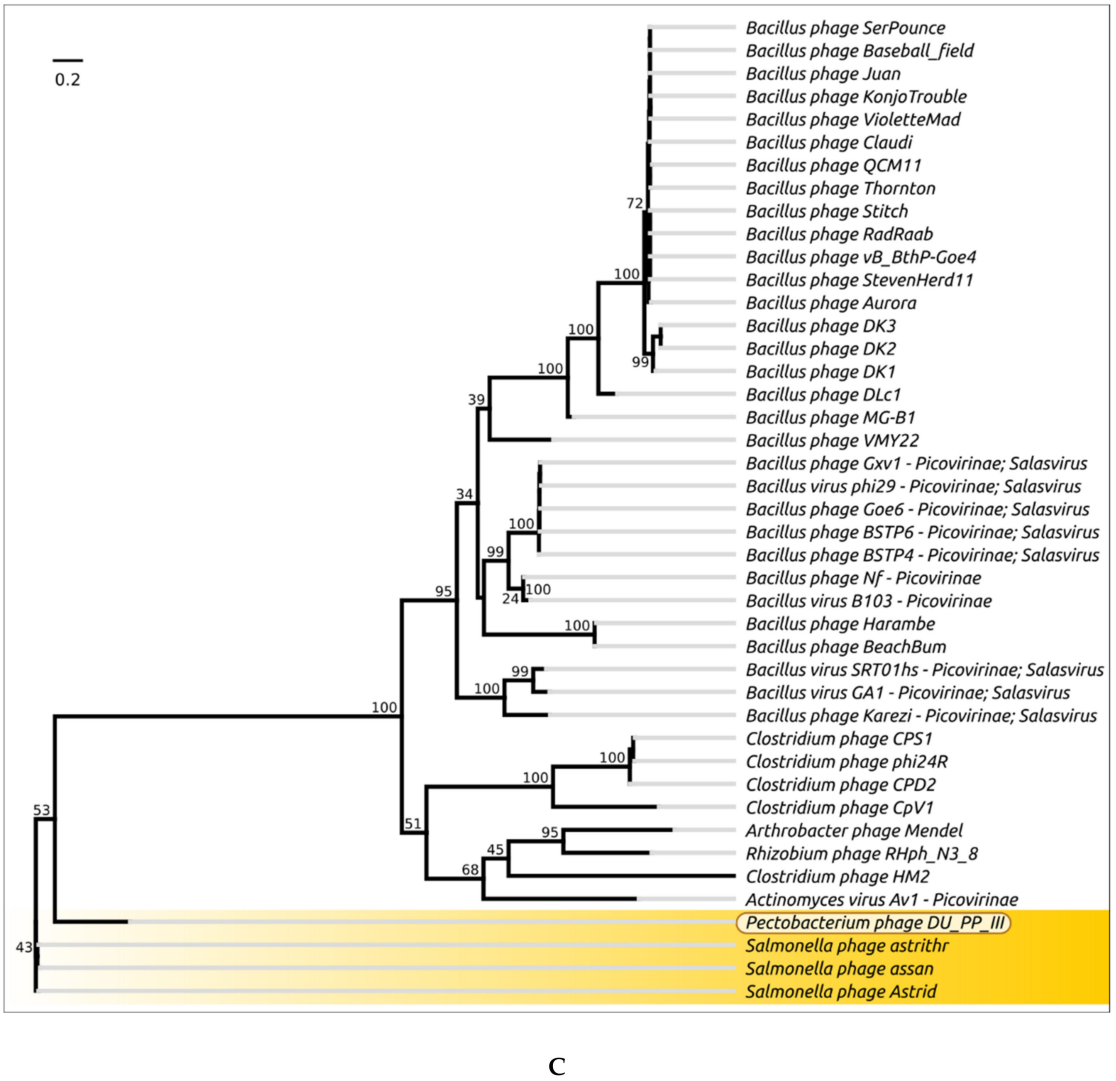

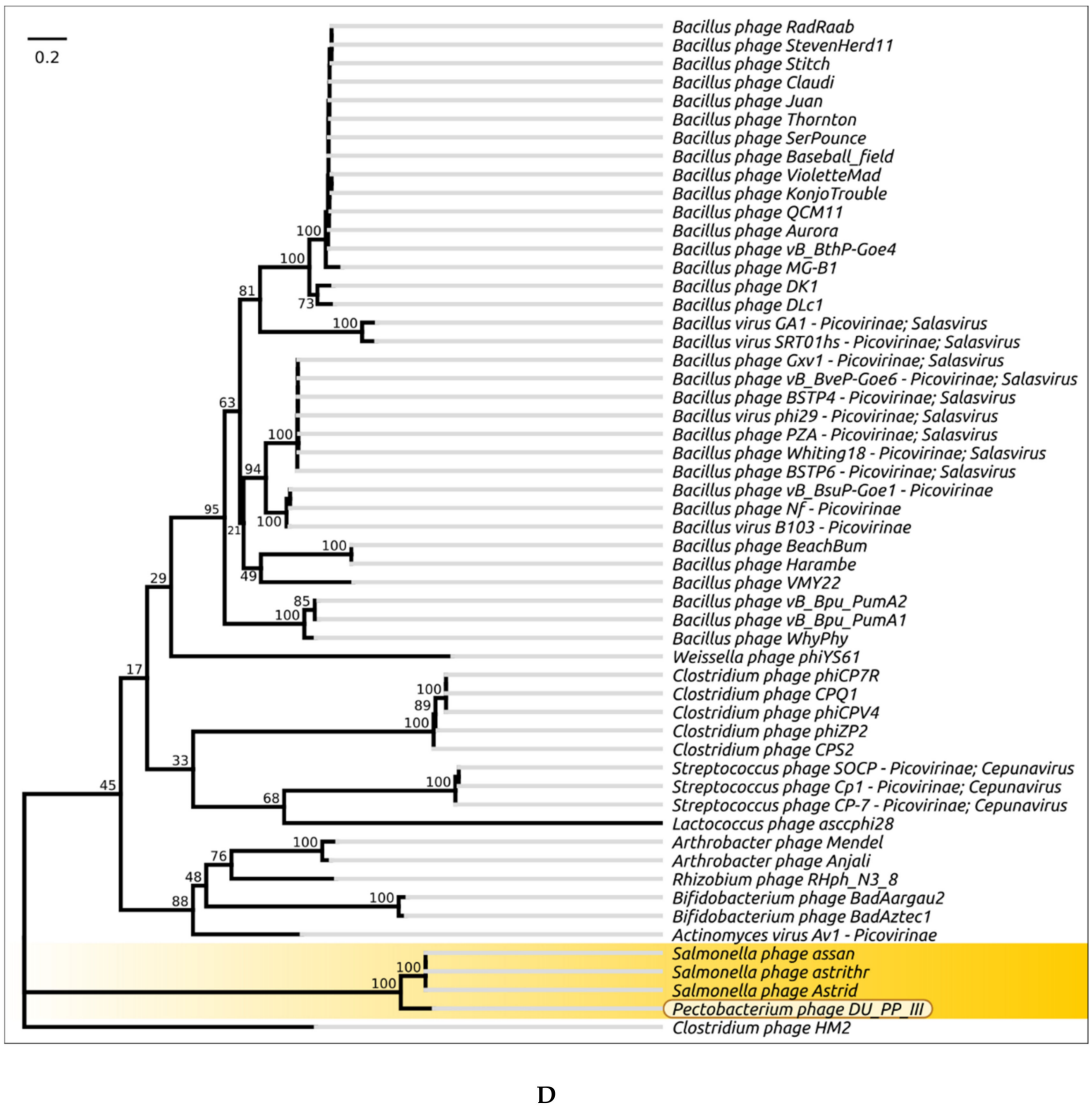
(**A**)**.** The genetic map of *Pectobacterium* phage DU_PP_III. The genes are coloured according to their functions; (**B**)**.** Best-scoring tree constructed with RAxML based on the DNA polymerase amino acid sequences. Taxonomic classification was taken from NCBI sequence attributes and is shown to the right of the phage name. Bootstrap support values are shown above their branch as a percentage of 1000 replicates. The scale bar shows 0.2 estimated substitutions per site and the tree was unrooted; (**C**). Best-scoring tree constructed with RAxML based on portal protein amino acid sequences. Taxonomic classification was taken from NCBI sequence attributes and is shown to the right of the phage name. Bootstrap support values are shown above their branch as a percentage of 1000 replicates. The scale bar shows 0.2 estimated substitutions per site and the tree was unrooted; (**D**). Best-scoring tree constructed with RAxML based on DNA polymerase amino acid sequences. Taxonomic classification was taken from NCBI sequence attributes and is shown to the right of the phage name. Bootstrap support values are shown above their branch as a percentage of 1000 replicates. The scale bar shows 0.2 estimated substitutions per site and the tree was unrooted.

The phage DU_PP_III, isolated from *Pectobacterium* species, possesses the smallest genome among the *Caudovirales* bacteriophages, submitted to NCBI GenBank. It has a linear genome with a GC-content of 37.4%, containing inverted terminal repeats of 219 bp in length. The genome contains 16 ORFs but no tRNA genes, and appears to contain neither integrase nor excisionase genes. Unfortunately, this interesting phage has not yet been comprehensively studied. The phage DU_PP_III genome was reannotated with the assistance of Prokka [163], using custom databases made with BLAST tools [164] and Prokka default databases. The open reading frames (ORFs) were predicted with Prodigal 2.6.1 [165], Glimmer 3.02b [166] and Geneious Prime 2021.2.2 [53] and manually validated and curated. The prediction of functions of the encoded proteins was carried out by means of a homology search and HMM-HMM comparison. The homology search was made using BLAST and the NCBI non-redundant (nr/nt) database and custom databases made with BLAST using GenBank phage sequences. The functions of proteins were assigned by comparison with known homologs. A HHM motif search was conducted using Phyre2 [167] and the HHpred server (PDB_mmCIF70, SCOPe70_2.07, ECOD_ECOD_F70, and UniProt-SwissProt-viral70 databases) [168,169] and functions were assigned by comparison with similar proteins with a threshold of 95% Phyre2 confidence or HHpred probability. The presence of tRNA coding regions was checked with tRNAscan-SE [170] and ARAGORN [171]. The scheme of the genome is presented in Figure 12A. Phylogenetic analyses were conducted on DNA polymerase (DNAP) (Figure 12B), encapsidation ATPase protein (terminase) (Figure 12C) and the portal (collar) protein (Figure 12D). A VIRIDIC intergenomic similarity analysis was carried out using 43 genomes of phages found by the BLAST search using the DNAP sequence (Supplementary Figure S14). These studies have demonstrated that DU_PP_III is related to phages Astrid, Astrihr and Assan [172] with similar genome size, infecting *Salmonella*, also belonging to the Enterobacterales. The intergenomic similarity of phage DU_PP_III and those *Salmonella* phages is about 40% and DNAP, the encapsidation protein and the portal protein of phage DU_PP_III are grouped with homologs of *Salmonella* phages in distinct clades.

The primary sequence and HMM-HMM motif comparisons indicated similarities of DU_PP_III proteins to proteins of *Bacillus* phage ϕ29 and its close relatives [173,174]. The HHpred analysis results indicate the strong structural similarity of seven DU_PP_III proteins to proteins of phage ϕ29 and its relatives. Particularly, the DU_PP_III DNA polymerase demonstrates similarities on approximately 95% of its length. In addition, the HHpred analysis shows the high level of similarity of the DU_PP_III DNA polymerase to DNA polymerases of *Streptococcus* phage Cp-1, *Enterobacteria* phage PRD1, adenoviruses and Acidianus bottle-shaped virus (ABSV). DNA polymerase of some plasmids, e.g., *Neurospora intermedia* mitochondrial linear plasmid Kalilo, also demonstrates a high level of similarity to the DNAPs of viruses listed above. These findings are very interesting, since ϕ29-like phages, phages Cp-1, RBD1 adenoviruses, ABSV and plasmid Kalilo employ protein primers (so-called terminal proteins, TPs) during DNA replication [174,175,176,177]. Furthermore, their genomes contain inverted terminal repeats and their 5’-ends are covalently bound with TPs. It is possible that phage DU_PP_III replication also involves the protein-primed mechanism.

Another interesting feature of phage DU_PP_III, which needs more detailed study, might be the use of packaging RNA (pRNA) for genome packaging. In ϕ29 and some other related phages, the DNA packaging motor includes the head–prohead RNA-ATPase complex acting as a stator and the connector as a ball-race [175,178]. According to the HHpred analysis, the encapsidation proteins of phages DU_PP_III and ϕ29 are similar along almost their entire length and the collar proteins are similar along 85% of the ϕ29 collar protein length.

Further research into DU_PP_III and related phages can contribute to understanding the details of viral evolution.

## 5. Morphotype *Siphoviridae*

Siphoviruses have a long and flexible tail which is used for adsorption, host cell wall perforation and delivery of the phage genome inside the infected cell. Classic representatives of this morphotype, such as phages λ, HK97and T5, have served as model objects for the study of viral replication, transcription, assembly, genome packaging, lysogenic conversion of the bacterial host and phage–receptor interactions. Siphoviruses are numerous and abundant in nature [179] and their genomes are prevalent in databases [12], albeit mostly as a part of metagenomes, being insufficiently annotated and studied. In general, Siphoviruses can be considered to have been the focus of few studies, compared to Myoviruses and Podoviruses. Besides model phages infecting *E. coli* and phages infecting essential Lactobacteria, few *Siphoviruidae* have been investigated in detail. A possible reason is that most phages of this type are known to be temperate, bearing genes for lysogeny in their genomes, thus having little potential for the purpose of phage therapy/biocontrol. However, highly virulent Siphoviruses are also known. The past decade has provided detailed structural insights into the process of Siphoviral adsorption and the initial steps of infection. Carbohydrates and membrane proteins of bacterial cells may serve as a primary receptor for phage adsorption [180], and concerted action of a tail tip protein, tape measure protein and tail-associated lysozyme is necessary for phage DNA injection [180,181,182]. Accessory tail fibres or smaller receptor-binding proteins contribute to phage binding specificity [180,183]. Therefore, the composition of the adhesion device may be a distinguishable hallmark of a particular phage taxon.

A literature search found reports on *Siphoviridae* bacteriophages infecting *Pectobacterium* and *Dickeya* spp. [184,185]. The morphology and biological properties of these phages were studied and some of them were used in *in vivo* experiments to assess their therapeutic potential. However, only a few of them were sequenced and characterised genomically. 

### 5.1. The Demerecviridae Family

Family *Demerecviridae* was created to replace the genus *Tequintavirus*. The genus was established in 2015, grouping bacteriophages related to classic *E. coli* phage T5. T5-like phages have uniform dimensions, with an 80–100 nm isometric capsid and a 200–250 nm tail with fibres on the distal tip. The genomes are 105–125 kb in length, with long terminal repeats, although terminal redundancy is not obvious and many phages are annotated as circular genomes. The genomic DNA of the phages has nicks on one of the strands. This feature was considered to be unique for T5-like viruses [186], until it was found in some Podophages [187]. Genomes usually encode more than 20 tRNAs. Despite many common features, the phages are diverse, and based on DNA and protein sequence relatedness, the members of the newly-formed family are divided into three subfamilies and nine separate genera. Phages infecting SRP are assigned to the subfamily *Mccorquodalevirinae*, comprising two genera, *Hongcheonvirus* and *Myunavirus*, each represented by only one type of phage genome. No detailed study of the biological features of these phages is available, so all information must be derived from the genome sequence. 

Bacteriophage DU_PP_V was first isolated in South Korea, (Hongcheon, the location of isolation being assigned as the name of the genus), using a non-specified *Pectobacterium* sp. as the host bacterium. The genome is 106,185 bp, encoding 127 proteins and 22 tRNAs (GenBank accession number NC 047887). The representative of the neighbouring *Myunavirus* genus, *Pectobacterium* phage My1, which also originated from South Korea [42], shares 59.8% of its DNA sequence identity with DU_PP_V. The genome of My1, of 122,024 bp, encodes 149 proteins and 20 tRNA. Both genomes have an arrangement of genes and promotors typical for T5-like phages. However, a difference exists in the predicted morphology of the adsorption apparatus. While My1 is predicted to contain L-shaped tail fibres similar to sensu stricto phage T5, the sequence of the DU_PP_V tail fibre is more similar to the fibres of the distant Siphophage T1. It is also worthy of note that a BLAST search reveals numerous homologs of DU_PP_V and My1 tail fibres in the genomes of *Pectobacterium* sp. So, although both phages are considered lytic and no obvious integration machinery is encoded in their genomes, it is possible that evolutionary predecessors of *Demerecviridae* have had a lysogenic infectious cycle. 

### 5.2. Unclassified Siphoviridae

The *Dickeya* phage Sucellus [131] and two similar *Pectobacterium* phages, MA11 and MA12 [188], are the only SRP *Siphoviridae* phages with complete or partial genomes presented in the GenBank database (Table 6).

**Figure 13:**
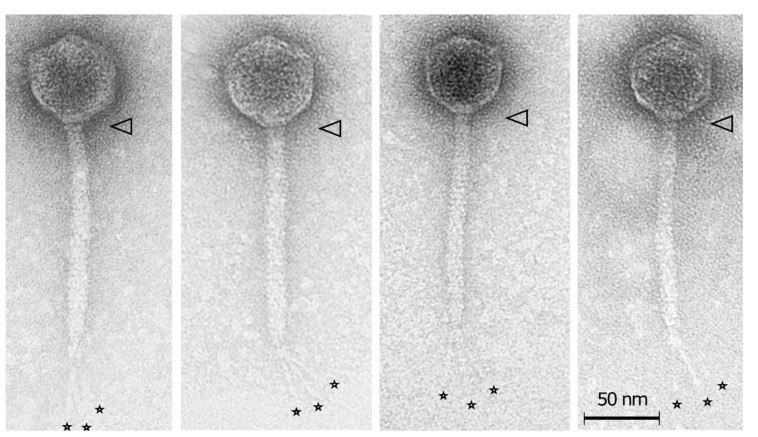
Transmission electron microscopy of *Dickeya* phage Sucellus [131]. It has a faint collar structure beneath the capsid (triangle) and three rigid, thin tail fibres attached to the conical end of Table 50 nm. The images were obtained with the kind permission of the authors and publishers of the cited papers.

*Dickeya* phage Sucellus (Figure 13) possesses a siphoviral morphology, with an isometric capsid of ~59 nm diameter, a tail ~137 nm in length, a faint collar structure beneath the capsid and three tail fibres of ~32 nm attached to the distal conical end of the tail [131]. The phage genome of 39,826 bp seems not to have genes for bacterial virulence or lysogeny [131]. Phylogenetic analysis has indicated that Sucellus has only distant relationships with other sequenced, unclassified *Siphoviridae* phages and should be considered a genomic singleton representing a novel genus [131].

*Pectobacterium* phages MA11 and MA12 represent another distinct group of siphoviruses related to the *Chivirus* genus of the *Siphoviridae* family [184]. Genomic analysis has not revealed the presence of lysogeny genes. The phage cocktail containing these phages has demonstrated a significant protective effect against natural soft rot infection in onions [184].

## 6. How Can the Knowledge of Phage Diversity Be Used in Practice?

Within the last decade, substantial progress has been made in the investigation of bacteriophages infecting SRP. The number of complete genomes deposited in accessible databases exceeds 100. Generally, the evolution of sequencing and data processing techniques suggests the further accumulation of available information in the future. Consideration should be given to the periodic undertaking of an inventory of available data on phage genomes in general [12,189], considering alignment with continually developing viral taxonomies. In the case of phages infecting a particular bacterial group, the analysis should include various aspects of the potential applications of these phages. The investigation of SRP phages does not contribute much to basic bacteriophage genomics. *Pectobacterium* and *Dickeya* are enterobacteria and no substantial difference is observed between phages of SRP and those infecting well-studied enteropathogens. However, even *Escherichia* phages have not been investigated comprehensively [188] and phage diversity continues to grow. Nevertheless, having an ample reference cohort enables an estimate of the inconsistencies between phages that have been studied and those that are yet to be discovered.

Applying standard technologies of phage isolation has resulted in a pronounced bias toward phages forming large, clear plaques, which are thus easier to purify and characterise. Clearly, the most prominent phages are *Autographiviridae* phages, which represent 63% of the SRP phages described and more than 80% of phages used in phage control experiments. Large and fast-growing plaques of these phages reflect the short period and high progeny typical for most studied *Autographiviridae*, which makes them suitable candidates for inclusion in phage cocktails to control plant diseases caused by SRP. Meanwhile, the plaques formed by Siphoviruses are usually small, and the isolation of Jumbo phages often requires a lower percentage of agar in double-layer techniques [190]. Thus, some phages may be overlooked, even if present in environmental samples studied.

This tendency reduces the potential diversity of the SRP phages that have been studied and hinders the applicability of phage control. Most SRP Podoviruses investigated use surface polysaccharides as primary receptors, thus enabling the target bacteria to alter their structure and form phage-resistant mutants. Phages can adapt their specificity to such mutants naturally, using horizontal gene transfer, as seen for different *Studiervirinae* phages using almost identical TSP [135], or through the acquisition of *Ackermannviridae*-like TSP by phage Sucellus. Due to the unified composition of their adsorption apparatus, Podophages and Myophages are often considered as a subject for directed gene engineering, adopting them for a broader range of host bacteria [191,192,193,194]. This approach is often criticised for the addition of a “genetically modified organism” status to the complicated goal of defining bacteriophages as therapeutic drugs [192]. Therefore, it seems more rational to browse and isolate SRP phages representing different taxons and using different molecules as receptors on the surface of the bacteria. The straightforward analysis presented in this work shows that even the limited diversity of the phages infecting *Pectobacterium* and *Dickeya* can employ a substantial variety of such receptors, and their inclusion in therapeutic cocktails to combat and prevent SRP infections in plants may increase treatment efficacy and reduce the formation of phage-resistant mutants of the pathogens.

## Figures and Tables

**Table 1:** Genomic properties of *Ackermannviridae* bacteriophages infecting SRP.

Phage	Isolation Host	GenBank Accession no.	Genome Size, kbp	% GC	ORFs	Reference
**Limestone**	*Dickeya solani*	HE600015	152.4	49.3%	201	[41]
**ϕD3**	*Pectobacterium* sp. and *Dickeya* sp.	KM209228	152.3	49.4%	190	[81]
**RC-2014**	*Dickeya* sp.	KJ716335	155.3	49.6%	196	[82]
**ϕJA15**	*D. solani*	KY942056	153.8	49.2%	198	[76]
**ϕXF4**	*D. solani*	KY942057	151.5	49.4%	195	[76]
**PP35**	*D. solani*	MG266157	152.0	49.3%	198	[80]
**Kamild**	*D. solani*	MH807812	152.6	49.2%	198	[75]
**Coodle**	*D. solani*	MH807820	152.5	49.1%	202	[75]
**Ds3CZ**	*D. solani*	MN788369	155.3	49.1%	201	[83]
**Ds5CZ**	*D. solani*	MN813048	154.7	49.1%	206	[83]
**Ds9CZ**	*D. solani*	MN813049	154.7	49.1%	204	[83]
**Ds16CZ**	*D. solani*	MN813050	152.8	49.2%	203	[83]
**Ds20CZ**	*D. solani*	MN813051	154.7	49.1%	202	[83]
**Ds23CZ**	*D. solani*	MN813052	149.4	49.4%	204	[83]
**Ds25CZ**	*D. solani*	MN813053	151.7	49.1%	194	[83]

**Table 2:** Genomic properties of *Vequintavirinae* bacteriophages infecting SRP.

Phage	Isolation Host	GenBank Accession No.	Genome Size, kbp	% GC	ORFs	Reference
**DU_PP_I**	*Pectobacterium sp.*	MF979560	145.0	50.3%	267	Direct Submission
**DU_PP_IV**	*Pectobacterium sp.*	MF979563	145.2	50.3%	268	Direct Submission
**PcCB7V**	*Pectobacterium sp. 7V*	MW367417	146.1	50.4%	269	Direct Submission
**ϕTE**	*P. atrosepticum*	JQ015307	142.3	50.1%	242	[112]
**vB_PatM_CB7**	*P. atrosepticum*	KY514263	142.8	50.1%	253	[113]

**Table 3:** Genomic properties of *Ounavirinae* bacteriophages infecting SRP.

Phage	Isolation Host	GenBank Accession No.	Genome Size, kbp	% GC	ORFs	Reference
**Arno162**	*P. atrosepticum*	MK290737	91.7	44.5%	146	Direct Submission
**Arno18**	*P. versatile*	MK290738	91.7	44.5%	147	Direct Submission
**Wc4**	*P. carotovorum* subsp*. carotovorum*	MN270891	92.0	44.7%	145	[115]
**Wc4-1**	*P. carotovorum* subsp*. carotovorum*	MN270892	92.0	44.7%	145	[114]

**Table 4:** Genomic properties of *Salmondvirus* bacteriophages infecting SRP.

Phage	Isolation Host	GenBank Accession No.	Genome Size, kbp	% GC	ORFs	Reference
**JA11**	*D. solani*	MH389777	255.4	44.5%	321	[126]
**JA13**	*D. solani*	MH460460	254.1	44.5%	323	[126]
**JA29**	*D. solani*	MH460461	253.3	43.8%	318	[126]
**JA33**	*D. solani*	MH460462	255.4	44.5%	321	[126]

**Table 5:** Genomic properties of *Schitoviridae* bacteriophages infecting SRP.

Phage	Isolation Host	GenBank Accession No.	Genome Size, kbp	% GC	ORFs	Reference
**vB_PatP_** **CB1**	*P. atrosepticum*	KY514264	76.0	48.7%	100	[130]
**vB_PatP_CB3**	*P. atrosepticum*	KY514265	76.2	48.7%	105	[130]
**vB_PatP_CB4**	*P. atrosepticum*	KY549659	76.6	48.6%	103	[130]
**Horatius**	*P. versatile*	MN812691	73.7	48.5%	102	Direct submission
**Nepra**	*P. atrosepticum*	MH059638	74.5	48.7%	92	[145]
**ϕA38**	*P. parmentieri*	KY083726	75.8	48.7%	97	[150]
**ϕA41**	*P. parmentieri*	KY769270	75.8	48.7%	97	[150]
**Possum**	*P. versatile*	MN812687	73.8	48.5%	102	Direct submission

**Table 6:** Genomic properties of *Siphoviridae* bacteriophages infecting SRP.

Phage	Isolation Host	GenBank Accession no.	Genome Size, kbp	% GC	ORFs	Reference
**Sucellus**	*D. dadantii*	MH059634	39.8	41.9%	100	[132]
**MA11**	*P. carotovorum*	MN518139	55.8	54.5%	38	[188]
**MA12**	*P. carotovorum*	MN692199	58.6	54.5%	38	[188]

## Supplementary Materials

The following are available online at https://www.mdpi.com/article/10.3390/microorganisms9091819/s1. Figure S1: Phylogenetic tree obtained with MrBayes, based on the major capsid protein amino acid sequences of SRP phages. Bayesian posterior probabilities are indicated near their branches. Taxonomic classification is shown to the right of the phage name. The scale bar shows 0.2 estimated substitutions per site and the tree was rooted to phage vB_PcaM_CBB. The chain length was 3,300,000, the burn-in length was 300,000, the subsampling frequency was 200 and the average standard deviation of split frequencies was 0.0078. Figure S2: Phylogenetic tree obtained with MrBayes, based on the terminase large subunit amino acid sequences of SRP phages. Bayesian posterior probabilities are indicated near their branches. Taxonomic classification is shown to the right of the phage name. The scale bar shows 0.2 estimated substitutions per site and the tree was rooted to phage vB_PcaM_CBB. The chain length was 3,300,000, the burn-in length was 300,000, the subsampling frequency was 200 and the average standard deviation of split frequencies was 0.0075. Figure S3: VIRIDIC generated heatmap of *Vequintavirinae* phages. The colour coding indicates the clustering of the phage genomes based on intergenomic similarity. The numbers represent the similarity values of each genome pair, rounded to the first decimal. Figure S4: Best-scoring tree found by RAxML, based on the terminase large subunit amino acid sequences of *Vequintavirinae* phages. Taxonomic classification was taken from ICTV and NCBI sequence attributes and is shown to the right of the phage name. Bootstrap support values are shown above their branch, as a percentage of 1000 replicates. The scale bar shows 0.2 estimated substitutions per site and the tree was unrooted. Figure S5: VIRIDIC generated heatmap of *Ounavirinae* phages. The colour coding indicates the clustering of the phage genomes, based on intergenomic similarity. The numbers represent the similarity values for each genome pair, rounded to the first decimal. Figure S6: Best-scoring tree found by RAxML, based on the terminase large subunit amino acid sequences of *Ounavirinae* phages. Taxonomic classification was taken from ICTV and NCBI sequence attributes and is shown to the right of the phage name. Bootstrap support values are shown above their branch as a percentage of 1000 replicates. The scale bar shows 0.2 estimated substitutions per site and the tree was unrooted. Figure S7: VIRIDIC generated heatmap of *Peatvirus*-related phages. The colour coding indicates the clustering of the phage genomes based on intergenomic similarity. The numbers represent the similarity values for each genome pair, rounded to the first decimal. Figure S8: Best-scoring tree found by RAxML, based on the terminase large subunit amino acid sequences of *Peatvirus*-related phages. Taxonomic classification was taken from ICTV and NCBI sequence attributes and is shown to the right of the phage name. Bootstrap support values are shown above their branch as a percentage of 1000 replicates. The scale bar shows 0.2 estimated substitutions per site and the tree was unrooted. Figure S9: VIRIDIC generated heatmap of *Autographiviridae* phages. The colour coding indicates the clustering of the phage genomes based on intergenomic similarity. The numbers represent the similarity values for each genome pair, rounded to the first decimal. Figure S10: VIRIDIC generated heatmap of *Schitoviridae* phages. The colour coding indicates the clustering of the phage genomes based on intergenomic similarity. The numbers represent the similarity values for each genome pair, rounded to the first decimal. Figure S11: VIRIDIC generated heatmap of *Kafunavirus* and related phages. The colour coding indicates the clustering of the phage genomes based on intergenomic similarity. The numbers represent the similarity values for each genome pair, rounded to the first decimal. Figure S12: Best-scoring tree found by RAxML, based on the terminase large subunit amino acid sequences of *Kafunavirus* and related phages. Taxonomic classification was taken from ICTV and NCBI sequence attributes and is shown to the right of the phage name. Bootstrap support values are shown above their branch as a percentage of 1000 replicates. The scale bar shows 0.2 estimated substitutions per site and the tree was unrooted. Figure S13: Best-scoring tree found by RAxML, based on the major capsid protein amino acid sequences of *Kafunavirus* and related phages. Taxonomic classification was taken from ICTV and NCBI sequence attributes and is shown to the right of the phage name. Bootstrap support values are shown above their branch as a percentage of 1000 replicates. The scale bar shows 0.2 estimated substitutions per site and the tree was unrooted. Figure S14: VIRIDIC generated heatmap of *Pectobacterium* phage DU_PP_III and related phages. The colour coding indicates the clustering of the phage genomes based on intergenomic similarity. The numbers represent the similarity values for each genome pair, rounded to the first decimal.Table S1: Genomic properties of 108 SRP bacteriophages published in the GenBank genome database, as of July 2021.
