# Supplementary figures and images for: Tailed Lytic Bacteriophages of Soft Rot Pectobacteriaceae
Source: Microorganisms. 2021 Aug 26;9(9):1819. doi: 10.3390/microorganisms9091819 (PMC8472413; doi:10.3390/microorganisms9091819)

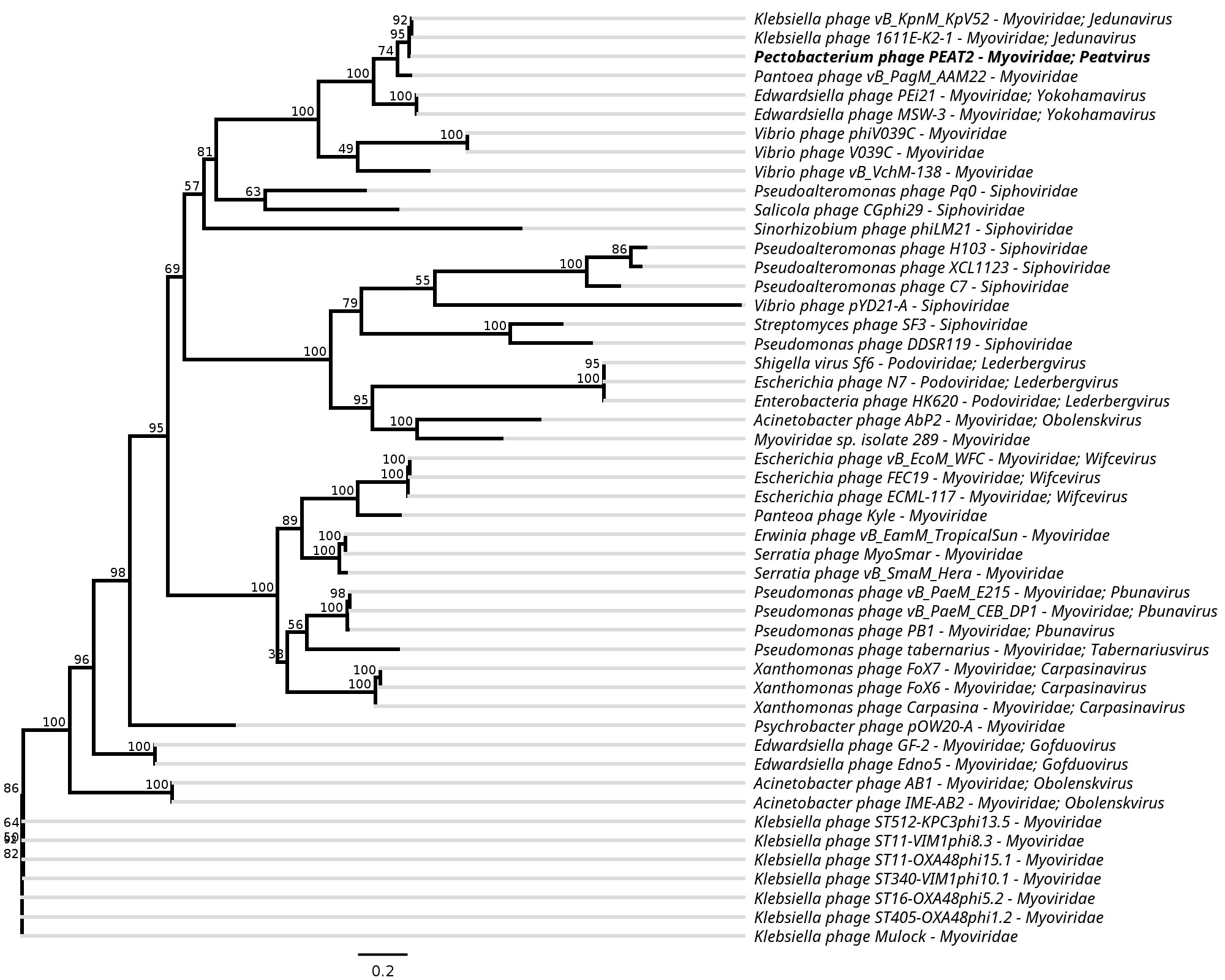

Supplement: Supplementary file 1 [file microorganisms-09-01819-s001.zip › Figure_S8.jpg]

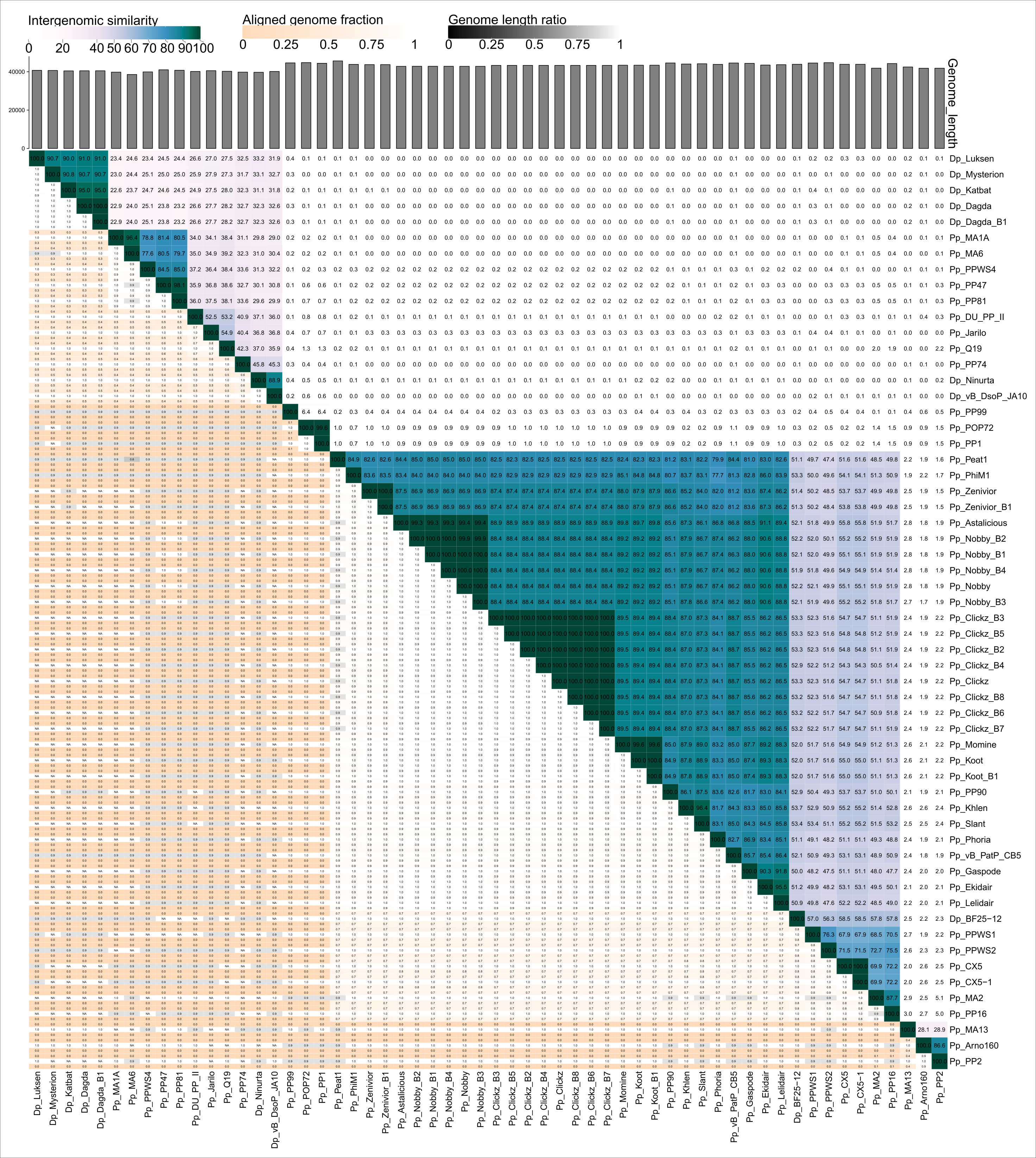

Supplement: Supplementary file 1 [file microorganisms-09-01819-s001.zip › Figure_S9.jpg]

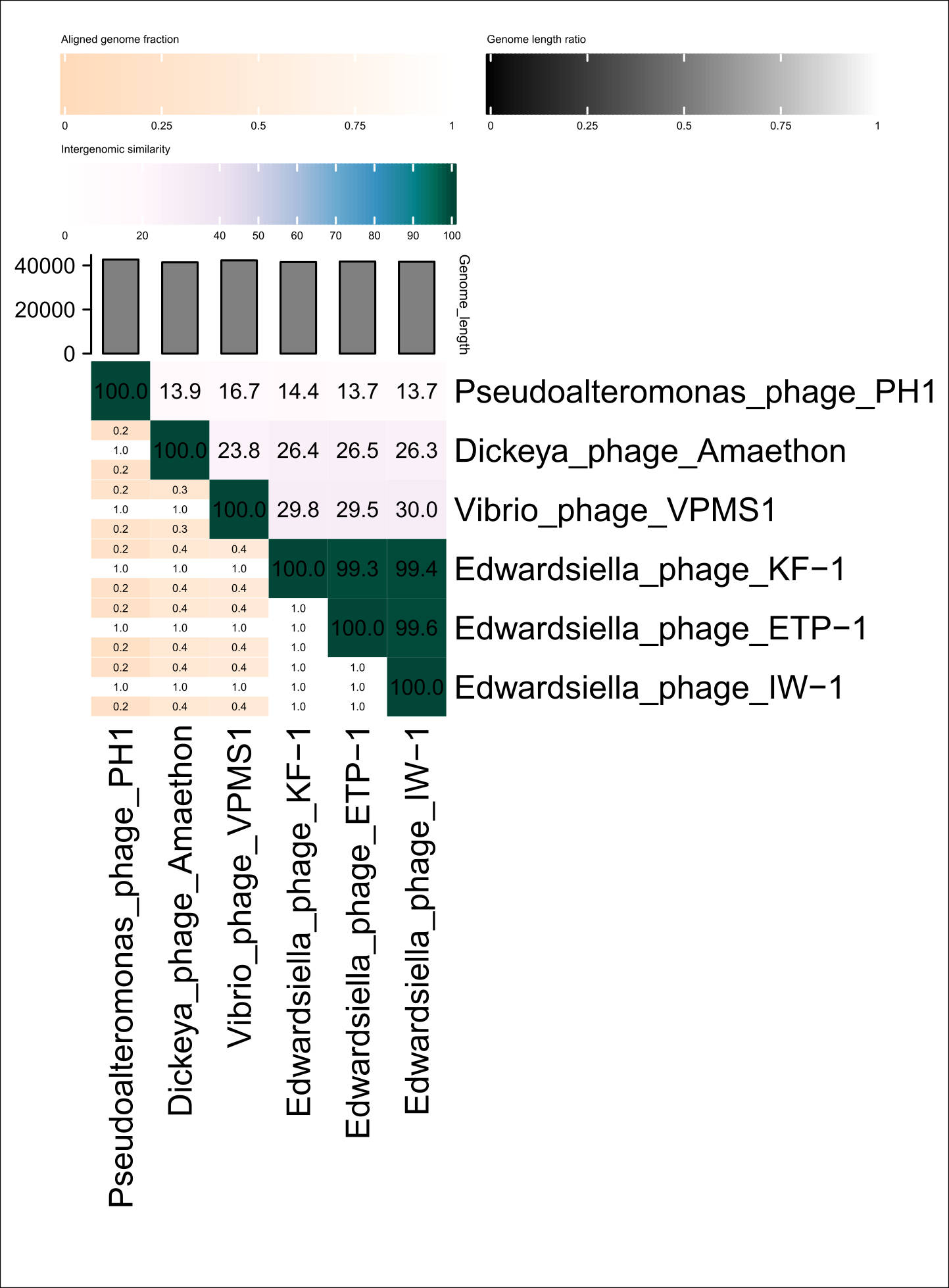

Supplement: Supplementary file 1 [file microorganisms-09-01819-s001.zip › Figure_S11.jpg]

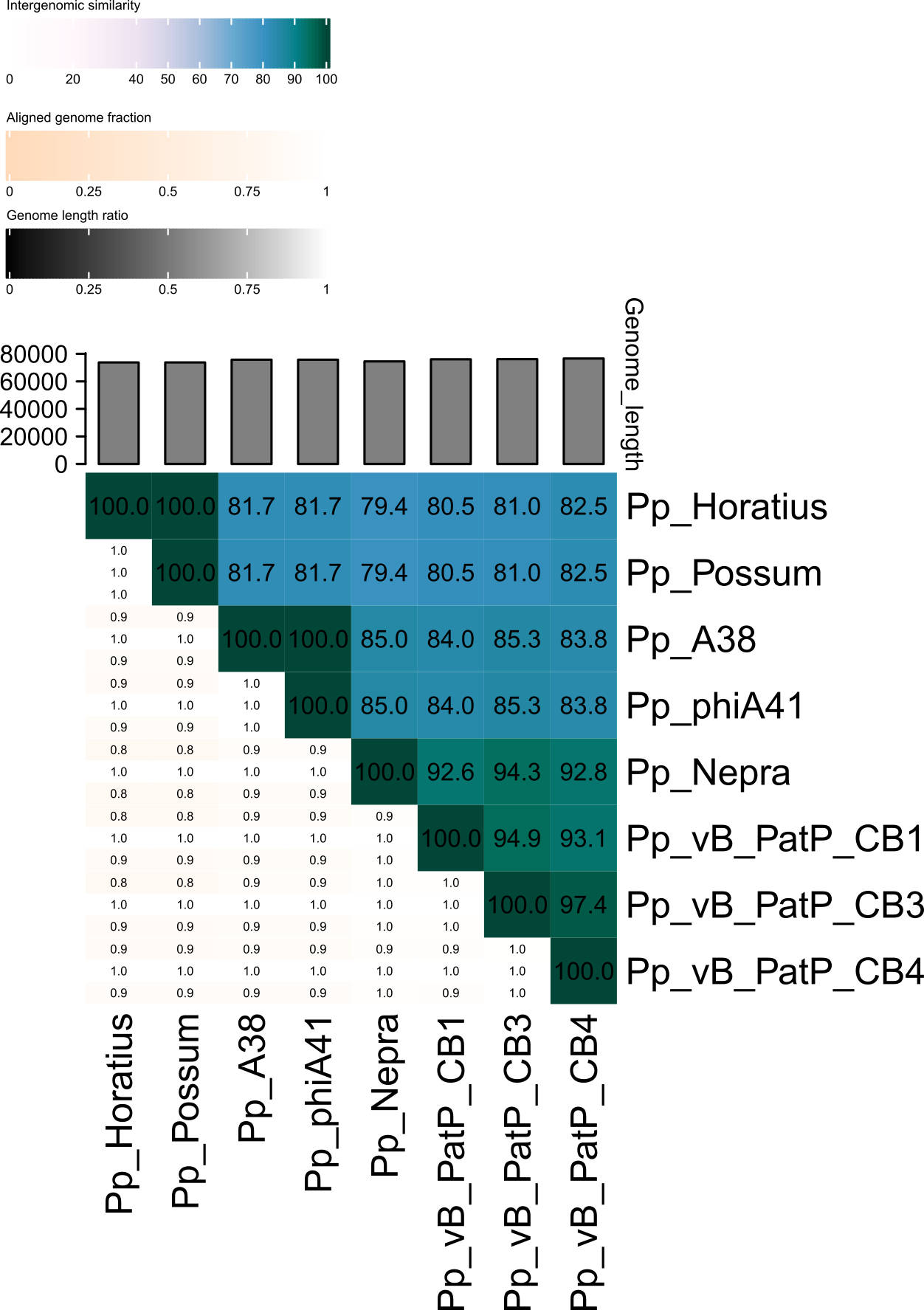

Supplement: Supplementary file 1 [file microorganisms-09-01819-s001.zip › Figure_S10.jpg]

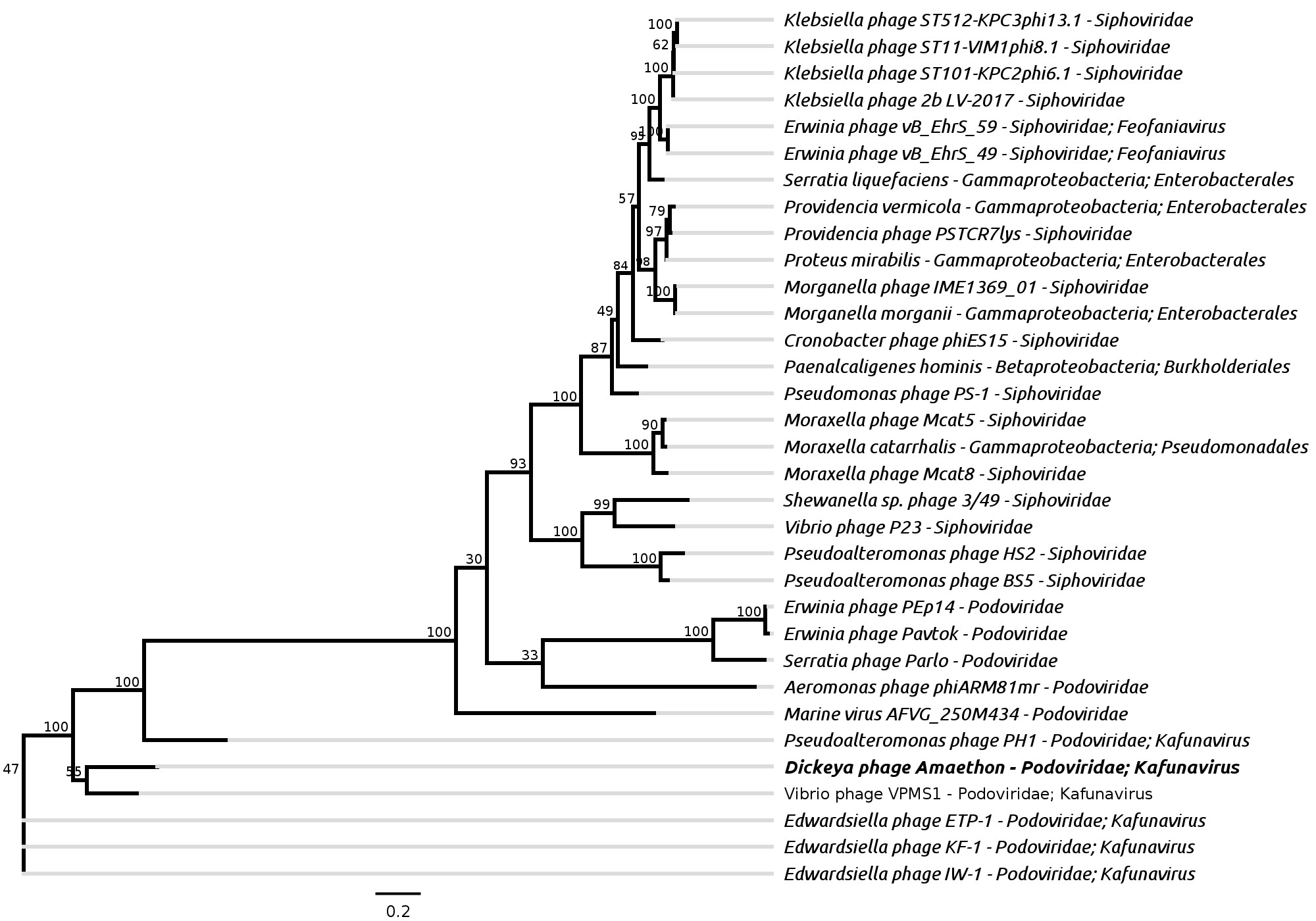

Supplement: Supplementary file 1 [file microorganisms-09-01819-s001.zip › Figure_S12.jpg]

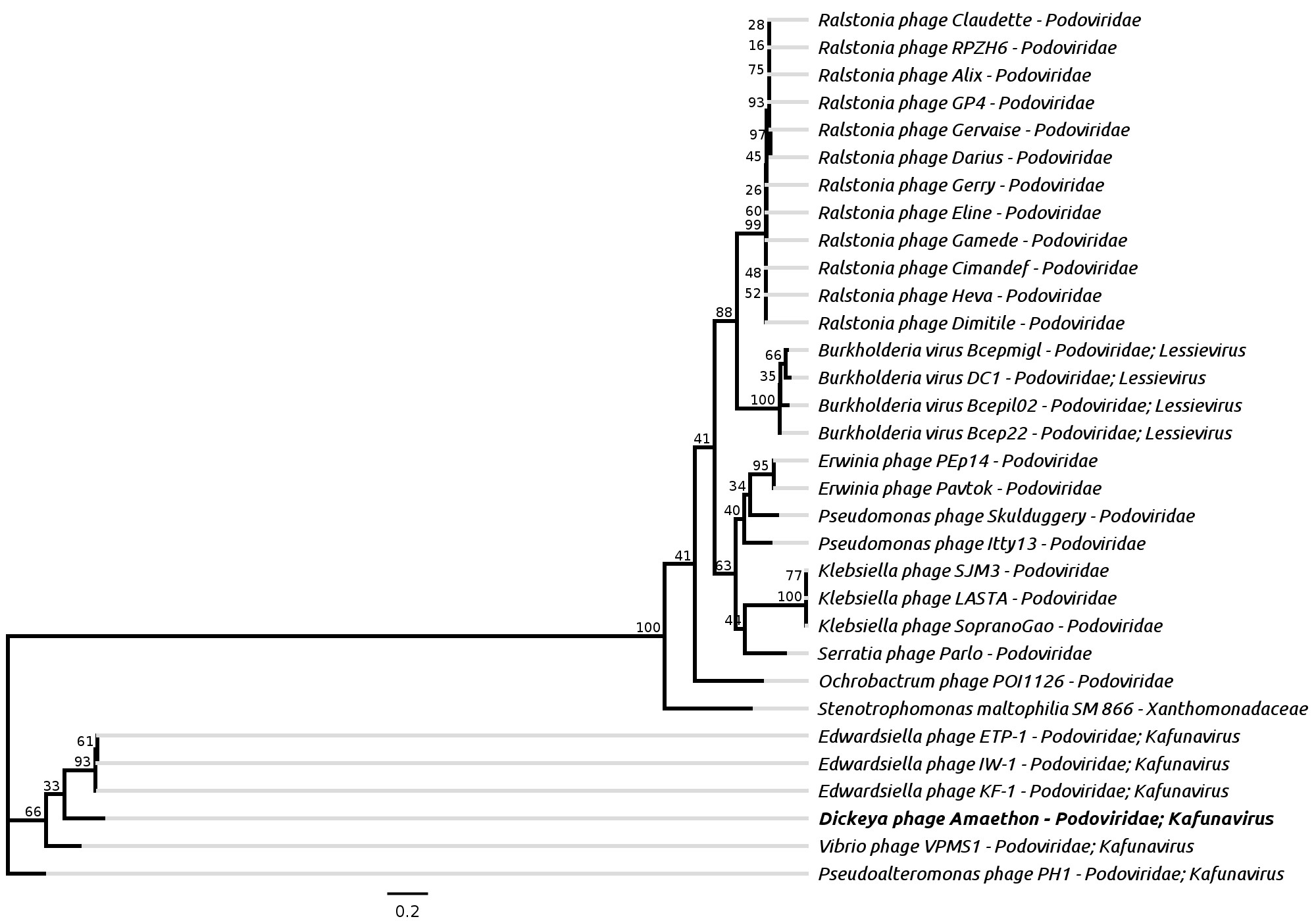

Supplement: Supplementary file 1 [file microorganisms-09-01819-s001.zip › Figure_S13.jpg]

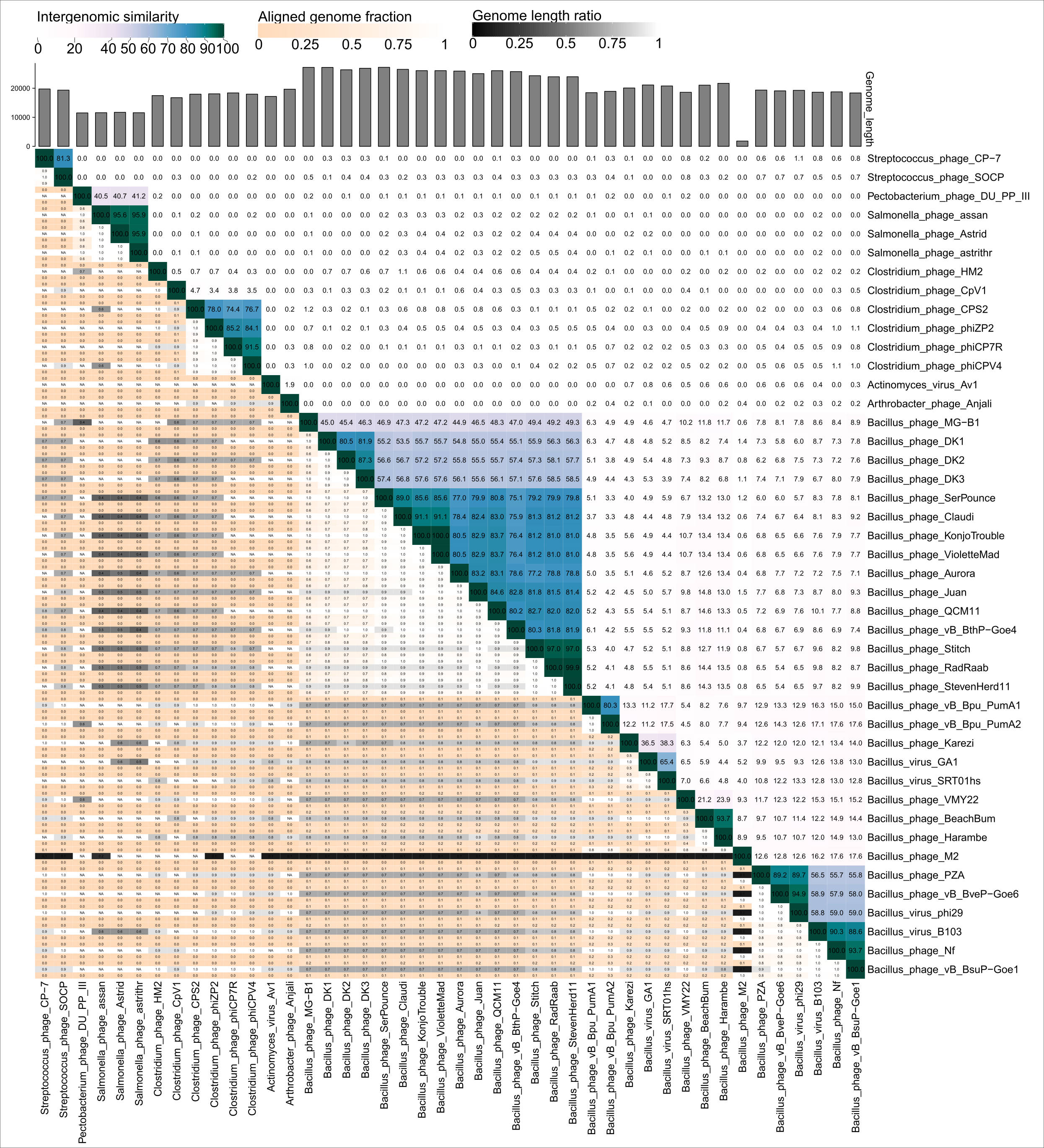

Supplement: Supplementary file 1 [file microorganisms-09-01819-s001.zip › Figure_S14.jpg]

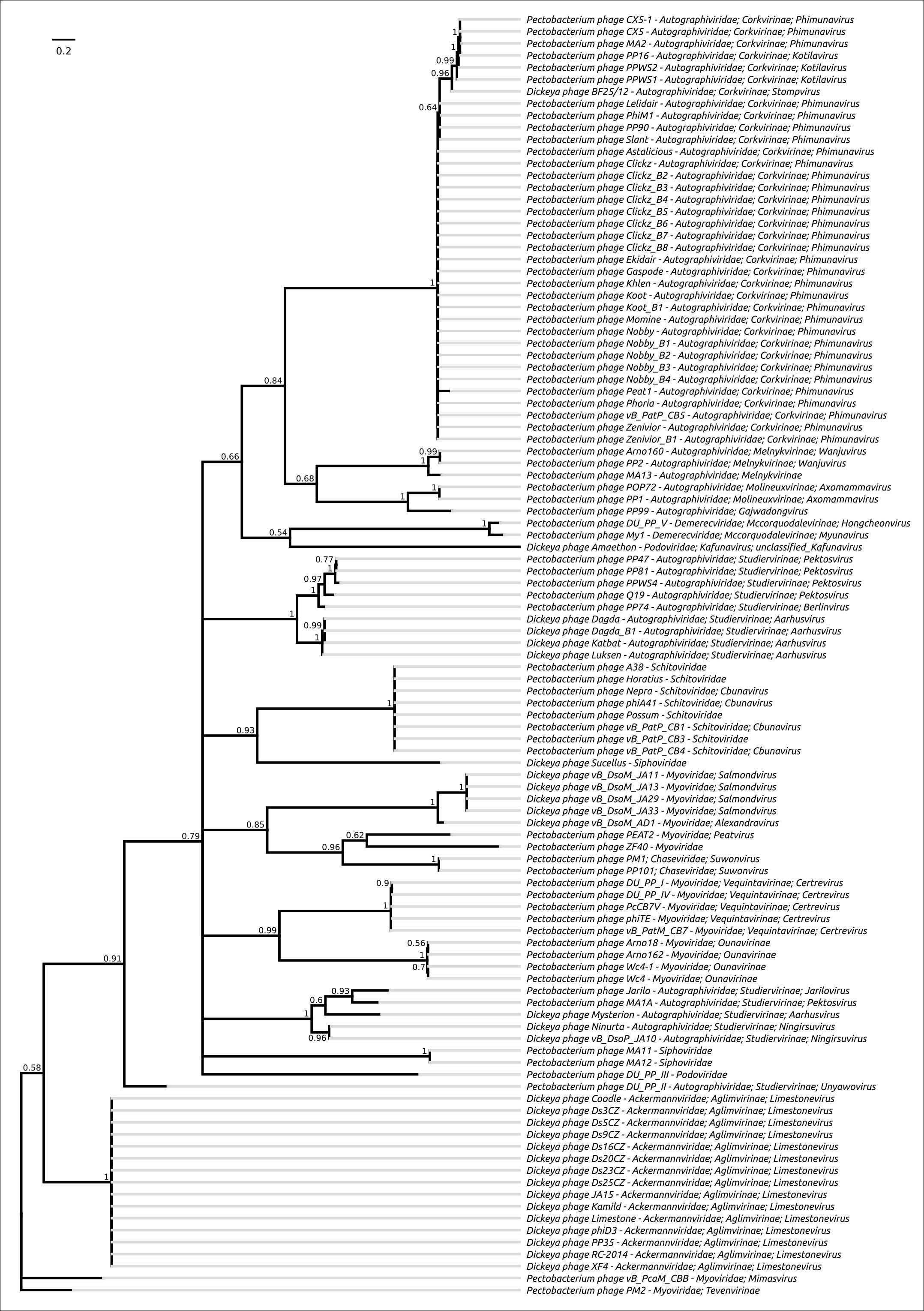

Supplement: Supplementary file 1 [file microorganisms-09-01819-s001.zip › Figure_S1.jpg]

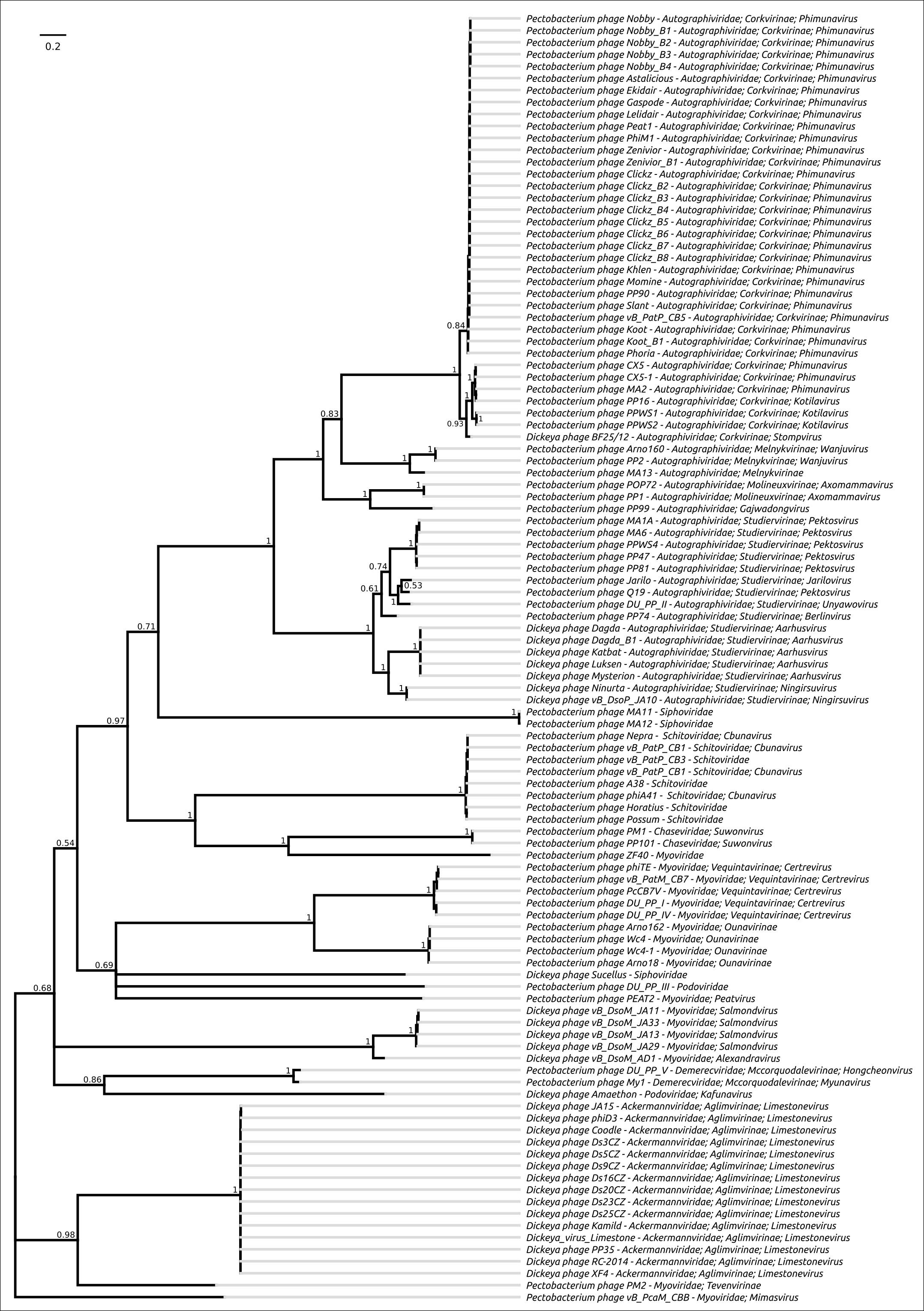

Supplement: Supplementary file 1 [file microorganisms-09-01819-s001.zip › Figure_S2.jpg]

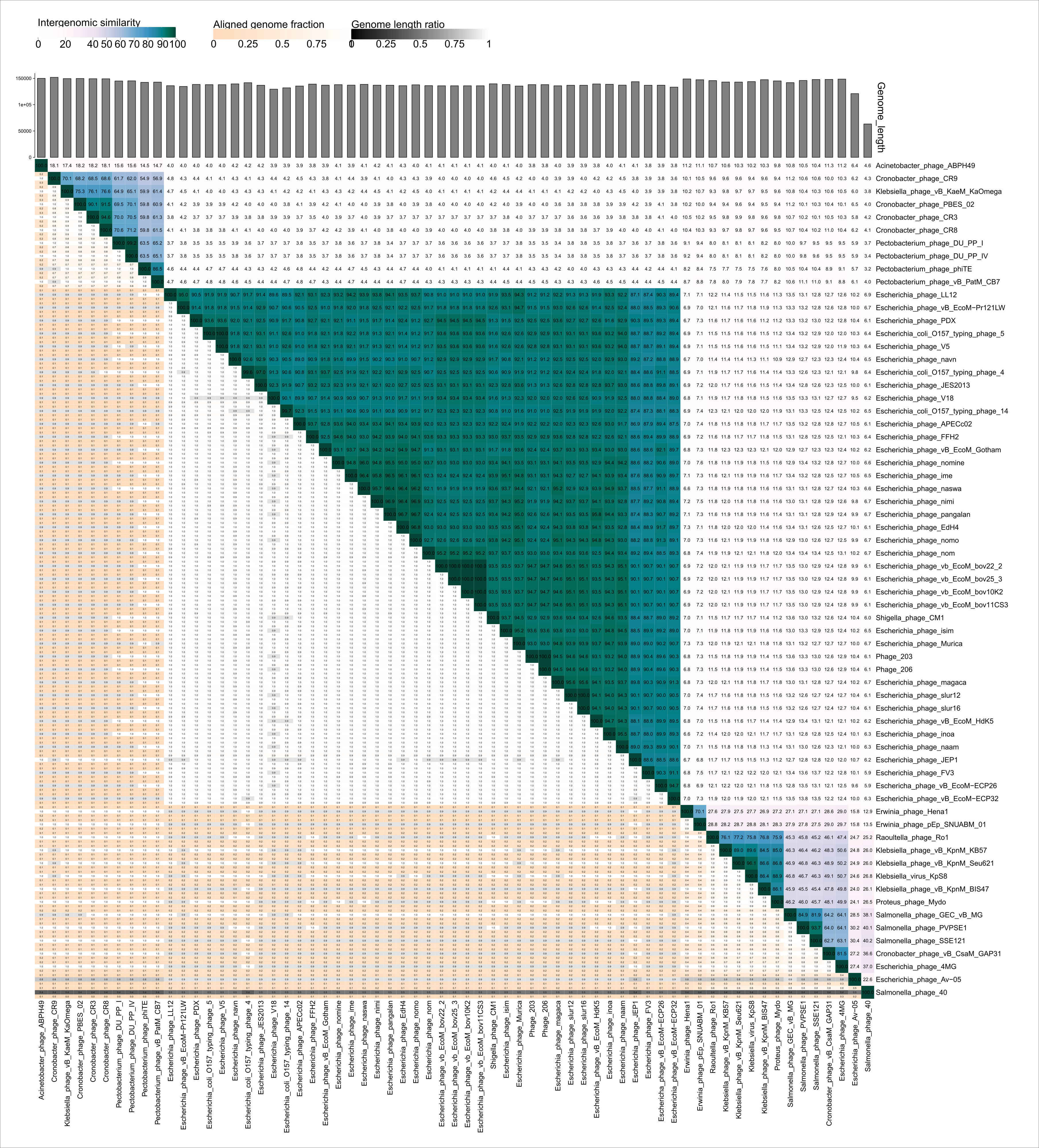

Supplement: Supplementary file 1 [file microorganisms-09-01819-s001.zip › Figure_S3.jpg]

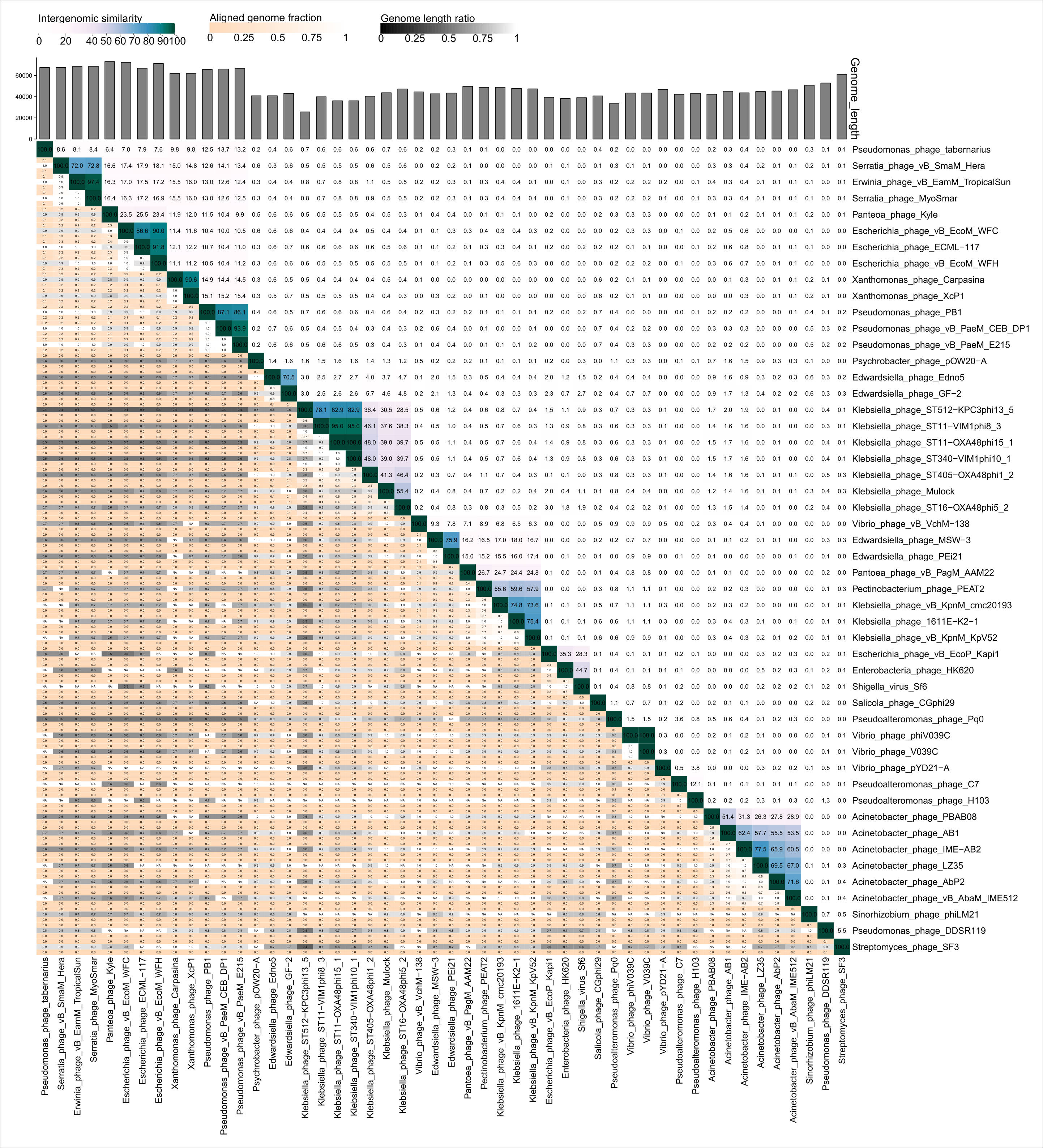

Supplement: Supplementary file 1 [file microorganisms-09-01819-s001.zip › Figure_S7.jpg]

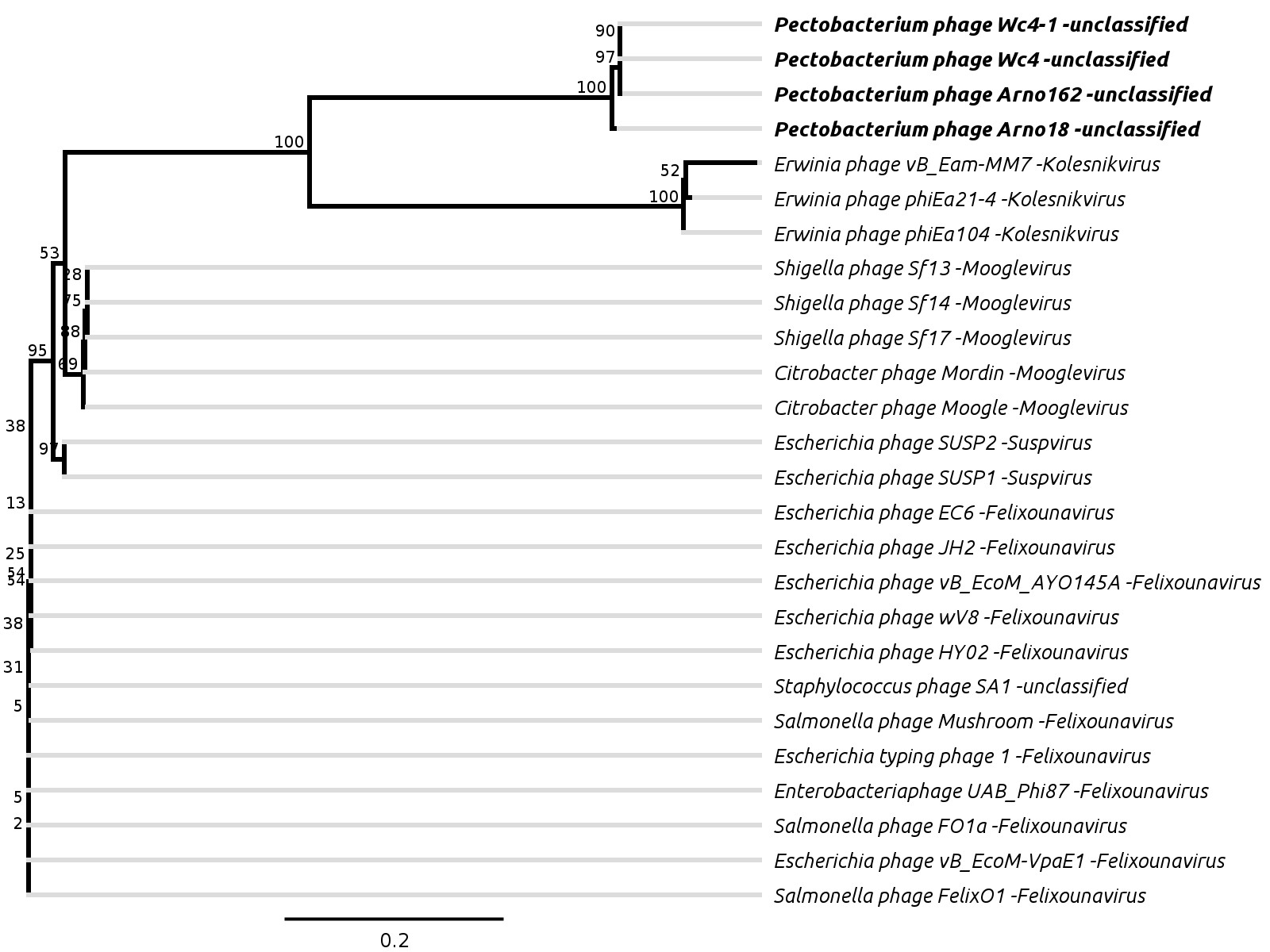

Supplement: Supplementary file 1 [file microorganisms-09-01819-s001.zip › Figure_S6.jpg]

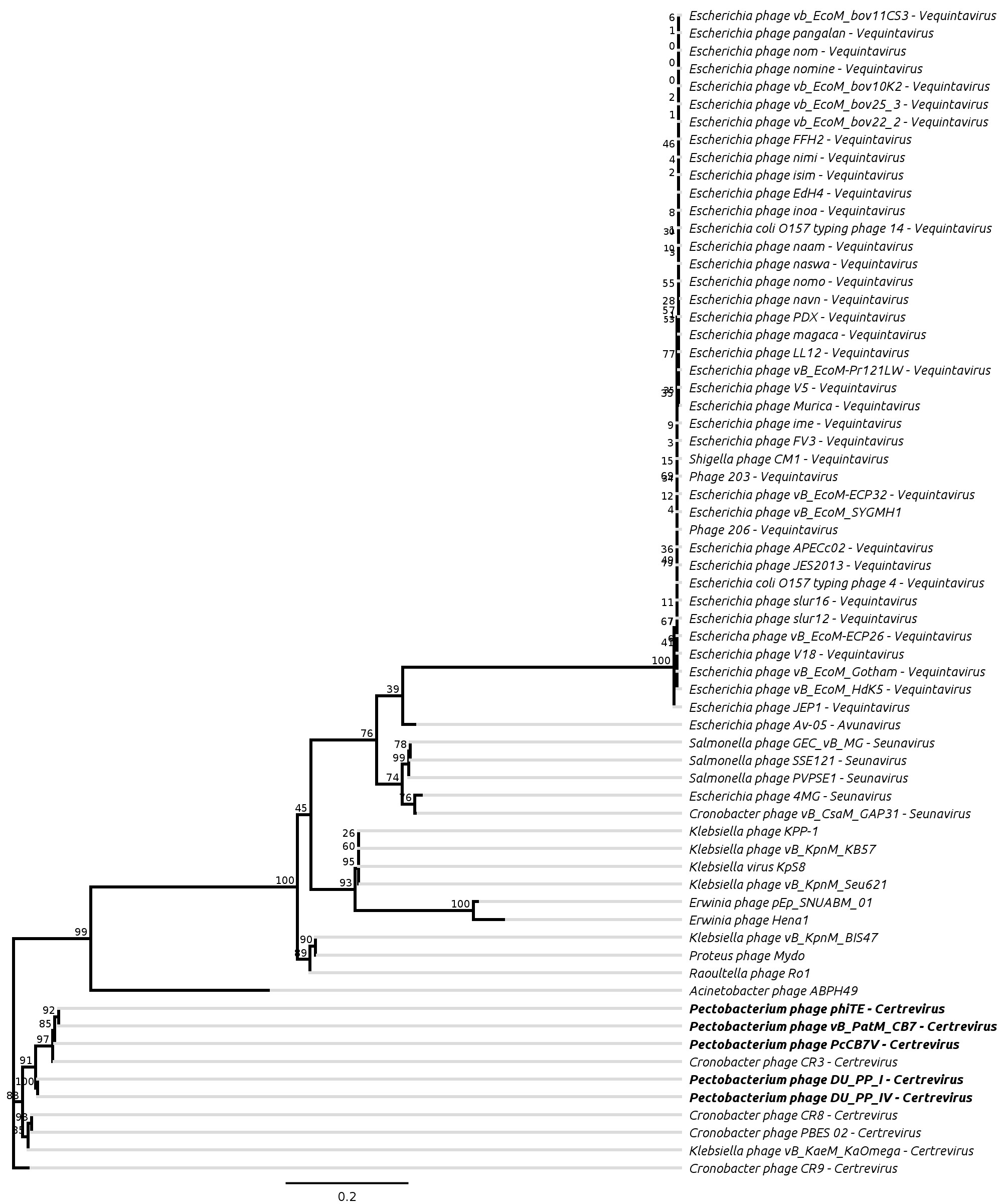

Supplement: Supplementary file 1 [file microorganisms-09-01819-s001.zip › Figure_S4.jpg]

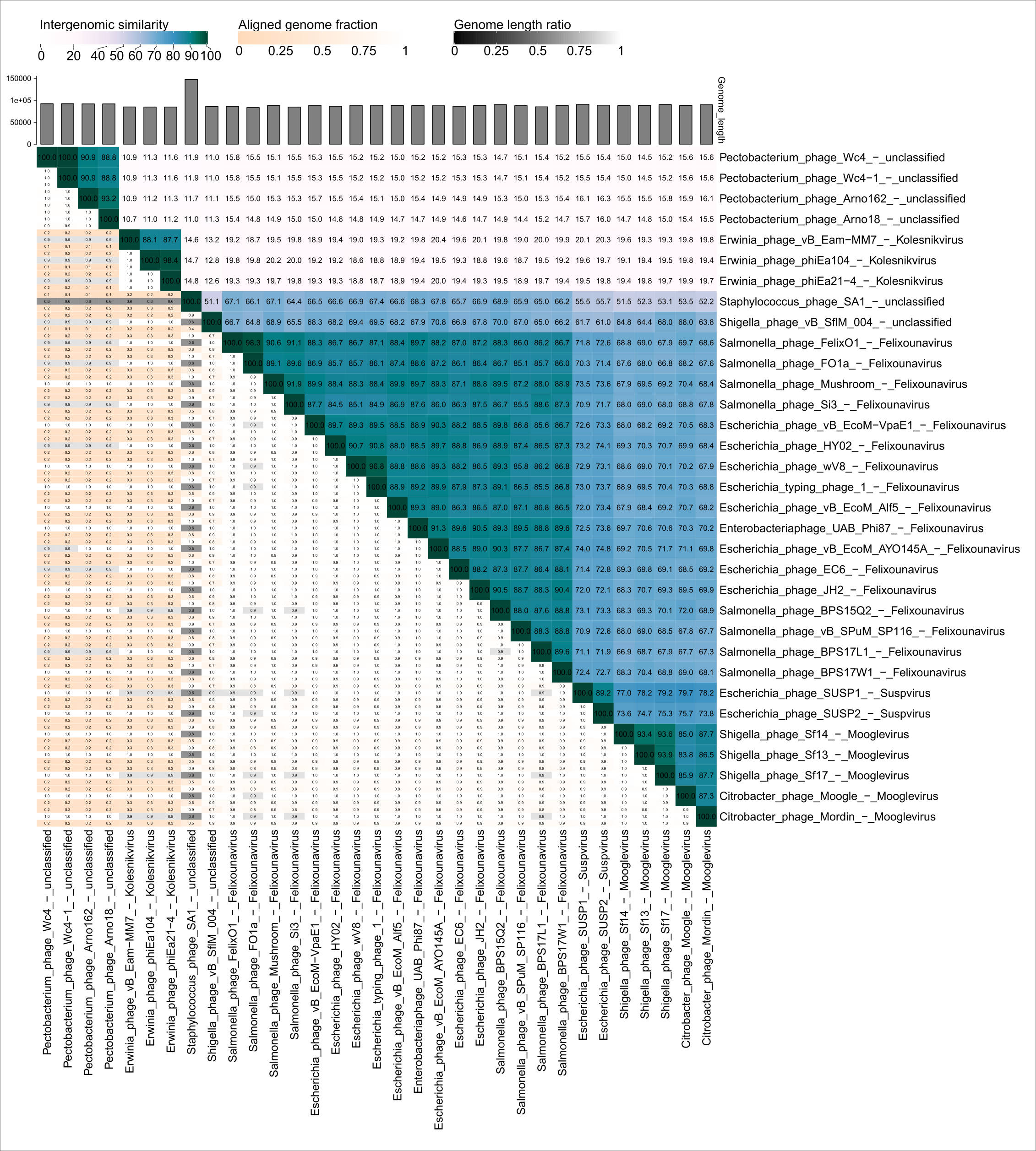

Supplement: Supplementary file 1 [file microorganisms-09-01819-s001.zip › Figure_S5.jpg]
